# Predicting Age From Behavioral Test Performance for Screening Early Onset of Cognitive Decline

**DOI:** 10.3389/fnagi.2021.661514

**Published:** 2021-07-12

**Authors:** Yauhen Statsenko, Tetiana Habuza, Inna Charykova, Klaus Neidl-Van Gorkom, Nazar Zaki, Taleb M. Almansoori, Gordon Baylis, Milos Ljubisavljevic, Maroua Belghali

**Affiliations:** ^1^College of Medicine and Health Sciences, United Arab Emirates University, Al Ain, United Arab Emirates; ^2^Big Data Analytics Center (BIDAC), United Arab Emirates University, Al Ain, United Arab Emirates; ^3^College of Information Technology, United Arab Emirates University, Al Ain, United Arab Emirates; ^4^Laboratory of Psychology, Republican Scientific-Practical Center of Sports, Minsk, Belarus; ^5^INSERM, COMETE, GIP CYCERON, Normandie University, UNICAEN, Caen, Research Unit: Aging, Health and Diseases, Caen, France; ^6^College of Education, United Arab Emirates University, Al Ain, United Arab Emirates

**Keywords:** aging, cognitive decline, biological age, psychophysiological tests, executive functioning, machine learning, cognitive impairment, neurodegeneration

## Abstract

**Background:** Neuronal reactions and cognitive processes slow down during aging. The onset, rate, and extent of changes vary considerably from individual to individual. Assessing the changes throughout the lifespan is a challenging task. No existing test covers all domains, and batteries of tests are administered. The best strategy is to study each functional domain separately by applying different behavioral tasks whereby the tests reflect the conceptual structure of cognition. Such an approach has limitations that are described in the article.

**Objective:** Our aim was to improve the diagnosis of early cognitive decline. We estimated the onset of cognitive decline in a healthy population, using behavioral tests, and predicted the age group of an individual. The comparison between the predicted (“cognitive”) and chronological age will contribute to the early diagnosis of accelerated aging.

**Materials and Methods:** We used publicly available datasets (POBA, SSCT) and Pearson correlation coefficients to assess the relationship between age and tests results, Kruskal-Wallis test to compare distribution, clustering methods to find an onset of cognitive decline, feature selection to enhance performance of the clustering algorithms, and classification methods to predict an age group from cognitive tests results.

**Results:** The major results of the psychophysiological tests followed a U-shape function across the lifespan, which reflected the known inverted function of white matter volume changes. Optimal values were observed in those aged over 35 years, with a period of stability and accelerated decline after 55–60 years of age. The shape of the age-related variance of the performance of major cognitive tests was linear, which followed the trend of lifespan gray matter volume changes starting from adolescence. There was no significant sex difference in lifelong dynamics of major tests estimates. The performance of the classification model for identifying subject age groups was high.

**Conclusions:** ML models can be designed and utilized as computer-aided detectors of neurocognitive decline. Our study demonstrated great promise for the utility of classification models to predict age-related changes. These findings encourage further explorations combining several tests from the cognitive and psychophysiological test battery to derive the most reliable set of tests toward the development of a highly-accurate ML model.

## 1. Introduction

The slowing of neuronal reactions and cognitive processes is a typical functional outcome of aging. However, the onset, rate, and extent of changes vary considerably from individual to individual. Furthermore, the breadth of cognitive function has led physiologists to describe cognitive performance in terms of domains of functioning; there is no single test that covers all domains, and batteries of tests are usually administered. Therefore, assessing the changes in the cognitive function throughout the lifespan of an individual is a challenging task. The best strategy is to study each functional domain by applying different conditions and behavioral tasks whereby the tests reflect the conceptual structure of cognition. This makes them suitable for both scientific research and practical studies. However, such an approach has limitations, which will be described in the article.

### 1.1. Structure of Cognitive Functioning and Cognitive Tests

The domains of cognitive function are hierarchical. The bottom of the cognitive construct is responsible for information input and refers to basic sensory and perceptual processes. The top of the construct is higher-order cognitive functioning. It maintains information processing that involves synthesis, accumulation, and retrieval from memory storage. The functions enable goal-driven behavior in an individual. The top-level elements are executive functioning (EF) and cognitive control. The domains are cross-dependent with the prevalence of top-down vs. bottom-up regulation. Broadly speaking, EF also encompasses cognitive control and exerts control over the use of more basic cognitive processes (Harvey, 2019).

Cognitive domains can be classified into memory, attention, language, and EF (e.g., reasoning and problem solving). EF is further classified into *inhibition, task switching, working memory updating, and information speed processing*, which are EF domains, or alternatively, cognitive subdomains.

Researchers consider dependent variables of executive functioning tests (EFTs) to be more sensitive to age-related changes than estimates of other types of cognitive functioning (Salthouse et al., 2003). Classical psychophysiological tasks are used to test EF target-specific functions. Assessments typically reflect subdomains of each ability, and careful combinations of tasks reveal patterns of performance that are consistent with a variety of neurological and neuropsychiatric conditions (Harvey, 2019). Typical limitations of the tasks are as follows:

- Despite the perfect usability of tests, many agree that practice effects influence follow-up performance on EFTs, which leads to potential overestimation of cognitive abilities in young people and underestimation of cognitive decline in older adults and patients (Overman et al., 2017).
- Because cognitive subdomains (both basic and higher-order) are closely interconnected, detecting changes that account for mutual compensation (e.g., speed-accuracy trade-off) can be difficult. However, such phenomena are common in physiology and may benefit permanent adjustments to variant conditions. Changing performance tactics may serve the surviving strategy. A solution was proposed by Beghali, where the Stroop switching task was modified by adding additional switching conditions to allow the assessment of overall EF using a single test (Belghali and Decker, 2019; Belghali et al., 2020).

Although age-related effects are more pronounced in EF than in other cognitive functions, the assumption that EF represents a distinct construct has received criticism (Salthouse et al., 2003; Salthouse, 2005). In a study of 261 cases, authors found “*only weak evidence for the existence of distinct constructs corresponding to EF or to aspects of executive control concerned with inhibition, updating, or time sharing,”* suggesting that researchers should not merely assume that variables reflect a particular hypothesized concept without relevant empirical evidence. To overcome such implications, we validated the Stroop switching card test (SSCT) in a recent study by comparing Stroop variables with the digital symbol substitution test, the digit span forward and backward test (DSFBT), the trail making test (TMT), and the classical Stroop test (Belghali et al., 2020).

Age-related cognitive changes are the key points of interest in interdisciplinary studies within the medical and behavioral sciences. Neurophysiologists, neurologists, and psychiatrists categorize cognitive processes into functional domains that have a hierarchical structure. The higher-order cognitive domains are cognitive control and EF, which account for the acquisition and processing of‘information.

Accurate assessment of cognitive status is important in neuroscience. To estimate cognition, EFTs are commonly used; however, there is no strong consensus that EFTs are reliable. In fact, some researchers have criticized the assumption that EF represents a distinct construct (Salthouse et al., 2003; Salthouse, 2005).

### 1.2. Psychophysiological Status and Tests, Functional Systems, Neural Hypernets

Psychophysiological tests (PTs) are alternative tools for assessing cognition and are also aimed at quantifying cognitive functioning domains, such as EF, information-processing speed, attentional control, and working memory. Commonly, a battery of PTs is composed of tests that cover all the constituents of cognition. However, they do not provide a summary assessment of whether the test results are associated with aging or disease. Instead, PTs provide an insight into an individual's psychophysiological status (PS). PS offers information on overall test performance, neuropathological changes, type of temperament, and trait features.

The idea of PS is closely linked to the *theory of functional systems*, which is a framework that describes the structure of an individual's behavior at the physiological and informational levels. Furthermore, it clarifies the cognitive architecture of an individual (Red'ko et al., 2004; Vityaev and Demin, 2018). According to the theory, goal-motivated activity comprises afferent synthesis, making a decision, and accepting the final result of an action (response selection). Thus, to estimate the PS of a person, clinicians should use a test battery that assesses all three components: the sensory component of a simple action, decision-making time, and response selection. A common EF test comprises these three elements.

All behavioral tests consist of consequent elements: *afferent synthesis* as a constituent of cognition, *decision-making* (an estimate of *information-processing speed*), and *response selection* (the core of attentional control). Additionally, PTs estimate the stability of regulatory system functions, which is also known as the level of *neuropsychological stability*.

Using a battery of PTs, neurophysiologists do not aim to target separate cognitive functions. Instead, they target physiological characteristics of the processes that underlie higher-order cognitive functions (e.g., EF). Sensorimotor response assessment in PTs is used to study the mechanisms of memory, information perception, and information processing, and by placing time limits or changing task complexity, it is possible to evaluate performance under various conditions. This allows psychophysiological compliance to be determined with some professional requirements. PTs have been validated as a cost effective and reliable tool to screen for professional maladjustment in sports and extreme professions (Li et al., 2019; Boichuk et al., 2020; Myroshnychenho et al., 2020). Unfortunately, clinical psychology does not meet the unconditioned cutoff criteria for major tests (Statsenko and Charykova, 2010).

Modern neurophysiological and neuropsychological studies have shown that specialized operations and systematic interactions of brain structures underlie cognition and behavior. The brain is structured and organized systematically and it includes projective, associative, integrative, and limbic-reticular function-specific systems. The systems closely interact with structures that are excited either simultaneously or alternatively. The functional elements are dispersed throughout the brain and separated, but not isolated, from each other. They maintain close cooperation, so that activation of one element can activate other elements. The basic unit of a functional system is a neuron, and a network of interconnected neurons is called a cooperative or cognitive group (cog). These networks contain an individual's innate and acquired knowledge and experience. The complete set of cogs forms a cognitome. The *theory of functional systems* has been further developed into the *theory of neural hypernets*, which describes the mind as a network in which the vertices are networks of functionally connected neurons. The representation of the mind as an organic and mathematical structure has fostered research applying experimental and theoretical physics, graph theory, and statistical mechanics approaches (Sudakov, 1997, 2015).

### 1.3. Onset of Cognitive Decline

Cognitive abilities (e.g., memory, thinking, and attention) begin declining from the age of 30. However, the rate of decline varies among individuals depending on genetics, lifestyle, regular mental activity, and somatic diseases. Compared with young and middle-aged adults, the elderly are more prone to lower mental performance, emotional lability, higher threshold of unconditioned reflexes, difficulties in developing conditioned reflexes, and fading of reflexes (Nelson and Luciana, 2001; Park and Gutchess, 2002). Because cognition reflects the integrated activity of the whole brain, cognitive impairment develops with focal and diffuse deterioration across various brain regions. The incidence of cognitive disorders increases with age, where 3–20% of people aged over 65 years have severe cognitive impairment (dementia) (Damulin, 2008). The incidence of mild cognitive impairment in the elderly ranges from 40 to 80% across different age groups (Larrabee and Crook, 1994). Usually, a diagnosis is made when an individual presents with evident cognitive deterioration and irreversible brain changes (e.g., dementia). Therefore, there is a need for improvements in diagnosis that allow the tracking of minor changes to detect early neurodegeneration. This will help to provide early prophylactic interventions and preventive measures to the elderly for sustaining a high level of intelligence.

## 2. Objectives

The overall aim of this study was to improve the diagnosis of early cognitive decline by applying a machine learning (ML) approach to psychophysiological and cognitive tests. We estimated the approximate age of onset of cognitive decline in a healthy population based on behavioral test performance and predicted individuals' age groups to compare with the their chronological age. Our objectives were:

1. To study the association between age and performance in psychophysiological and cognitive tests.
2. To estimate the onset of age-related decline in intellectual functioning.
3. To study sex differences in lifelong dynamics of the psychophysiological and cognitive test performance.
4. To develop a tool for identifying accelerated cognitive decline using the test results.

## 3. Materials and Methods

### 3.1. Methodology of the Study

*To address the first objective*, we assessed the relationship between age and test performance. To do so, we calculated Pearson's correlation coefficients. For each age group, the relationships between the continuous features were assessed using the Kruskal-Wallis test.

*For the second objective*, we studied the distribution of test performance values by age. Trendlines that approximate the distribution functions were determined with the least squares method to estimate second-order polynomial coefficients. The parabolic trendline functions were displayed using 95% confidence intervals, which were calculated using the bootstrap method. We developed a descriptive model of cognitive decline by comparing the polynomial regression function fits for the different tests. To find a possible onset of cognitive decline we assessed mean values and variance of tests results in age groups. For this we used descriptive statistics methods.

*To address the third objective*, we analyzed the patterns of the sex-specific features of lifelong performance dynamics of the psychophysiological and cognitive tests. We built ordinary least squares regression trendlines and expressed results as *IQR*, *mean*±*std* or number of cases, and their percentage out of the observed group. With Kruskal–Wallis test we assessed whether sex affected the impact of age on test performance (i.e., whether there was an interaction effect). To examine differences between the slopes and intercepts we used a *t*-test.

*The fourth objective* was multifold. We hypothesized that in normal aging there is a cutoff age from when cognitive decline begins. Some clustering techniques allow solutions to be built based on the number of clusters which can be predefined by the user. This allows one to test several possible divisions to determine the optimal model with clear separation of the identified groups.

*To achieve the first part* of the fourth objective of determining the age at which cognitive decline can be identified from test performance, we utilized a ML approach. We used an exploratory analysis by assessing the separability of datasets using unsupervised ML algorithms. We used clustering methods, such as Simple K-means (Arthur and Vassilvitskii, 2006), canopy (McCallum et al., 2000), expectation-maximization (Dempster et al., 1977), and GenClus++ (Islam et al., 2018). Testing different numbers of clusters based on performance allowed us to determine the possible onset of cognitive decline. Then we built pairwise distributions of each attribute by age. The battery of PT that we used resulted in a large number of dependent variables (e.g., time estimates and accuracy metrics). For the analysis, we employed the major tests results explained in section 3.2.1.

*For the second part* of objective four, we studied the informative value of the tests for detecting cognitive changes in the elderly. To enhance the performance of the clustering algorithms, we used feature-selection methods, which are designed to minimize overfitting and reduce the time needed for training, while increasing model performance metrics by eliminating less informative features from the dataset. We employed the genetic algorithm (Hall, 1998) and information gain attribute evaluation (Kononenko and Hong, 1997). The genetic algorithm retrieves the most relevant features, whereas information gain attribute evaluation-based ranker lists the attributes in descending order based on their informative value for the final prediction. These values are considered as a useful measure of feature importance in the final model decision.

*In the third, final part* of the fourth objective, we built an ML algorithm to predict the age group from an individual's cognitive test performance. This fulfills the final aim of detecting misclassified cases that are susceptible to accelerated brain aging based on cognitive status assessment. To build the desired solution, we used several binary classification algorithms, such as support vector machines (Platt, 1999) with linear and non-linear (radial basis function) kernels, Gaussian Naive Bayes (John and Langley, 2013), Bagging meta-estimator (Louppe and Geurts, 2012), an extra-trees classifier (Geurts et al., 2006), a random forest classifier (Breiman, 2001), and multilayer perceptron (Glorot and Bengio, 2010). Because of the relatively small size of the datasets, we used a stratified five-fold cross-validation technique to have confidence that the predictions will generalize to unseen data. To evaluate the performance of the predictive models, we generated a receiver operating characteristic (ROC) curve averaged over five folds. We also calculated mean sensitivity, specificity, balanced accuracy (BAC), and area under the curve (AUC) values with respect to class. These performance measures were suitable as the datasets were balanced across the age attribute. Finally, we determined the cases that were misclassified by the best predictive model. We used the confusion matrix and calculated false-positive (FP) and false-negative values (FN).

### 3.2. Datasets Description

#### 3.2.1. POBA Dataset

We used the dataset called *Psychophysiological outcomes of brain atrophy* (POBA; see section Acknowledgments). The methodology of the neurophysiological tests used for the dataset is well-defined and relevant to research on age-related functional changes. The accurate computerized assessment of PS was strongly aligned with the purpose of the study. The POBA dataset does not contain any complicated tests and comprises simple tasks that are suitable for those with different intelligence levels. The dataset consisted of 231 cases which included MRI examinations and psychophysiological testing results of people aged 4–84 years. Written patient or parental consent for minors for participation was obtained from each case. All participants were either patients who suffered from periodic headaches or were anxious about having organic brain pathology, or healthy participants who were examined at the beginning of their professional sports career. The exclusion criteria were as follows: organic brain pathology, mental disorder, or head injury. The dataset is available on demand (see section 8). A thorough description of the dataset has been previously published (Statsenko et al., 2020).

We have highlighted only the features used in this study to determine PT dynamics across the lifespan and for ML analysis. We used the following PTs:

1. *Simple visual-motor reaction* (SVMR): Reaction time (RT) is recorded for a single type of stimuli requiring an identical response. The result of the test is mean RT (SVMR_mean), which reflects the participants current functional state and indicates overall working capacity, type of temperament, and level of excitability of the central nervous system.
2. A type of*go/no-go test* with similar visual and motor components as the SVMR but with two types of stimuli that require different responses. For this reason, it is also called the *complex visual-motor reaction* (CVMR). The mean RT (CVMR_mean) correlates negatively with psychometric measurements of intelligence (Colman, 2015).
3. *Decision-making time* (DMT) is defined as the time taken for response selection. It is measured as CVMR_mean subtracted by SVMR_mean.
4. *Attention study technique*: To test attention, identical triggering stimuli are presented subsequently in different locations on a computer screen. The mean response time (AST_mean) reflects the level of attention to visual objects, stability, concentration of attention, speed of information processing, and work efficiency.
5. *Interference resilience technique*: In contrast to the previous task, this technique includes additional interfering objects (e.g., circles of different color and size) that overlapping each other and the targeted stimuli, which requires additional time for the participant to notice the triggering signal, and respond. The system calculates the average response time (IRT_mean).
6. The *time delay in responding to the targeted stimulus due to visual interfering objects* (TRVI) is the subtraction of AST_mean from IRT_mean (see Formula 2).
7. *Reaction to a moving object* (RMO) technique: A circle appears on the screen with one red and one green colored mark arranged radially. It becomes quickly filled with a yellow color in a clockwise direction from a starting point to the finishing line. The participant responds when the yellow sector passes through the red finishing mark. The result is measured as a mean value (RMO_mean) of the positive (time delays) and negative values (premature responses). A negative RMO_mean indicates a predominance of excitation of the central nervous system, whereas a positive RMO_mean indicates a predominance of inhibition of the central nervous system. Although RMO test results include a time parameter, the variable is an additional indicator of reaction accuracy (e.g., a delayed or proactive reaction).
8. *RT variability*: The dependent variables mentioned above measure the mean RTs calculated over 30 subsequent episodes of testing with varied time intervals. The standard deviation of RT conveys unique information beyond that offered by mean performance (Graveson et al., 2016). We analyzed the SD for each task as a separate dependent variable (SVMR_variance, CVMR_variance, AST_variance, IRT_variance, RMO_variance).
9. We used *wrist dynamometry* to measure the *maximum muscular strength* of the right (WDR_MMS) and left hand (WDL_MMS).
10. *Asymmetry coefficient* (AC) is calculated as the ratio of the maximum muscular strength of the wrists (see Formula 3). A study showed an association between the depth of the central sulcus (anatomic brain asymmetry) and the predominant use of the right or left hand for skilled and unskilled activities (Amunts et al., 2000). As anatomic brain asymmetry accounts for the functional asymmetry of the extremities, AC may reflect the difference in power between hands.

(1) $$\begin{array}{r} DMT=CVMR\text{\_}mean-SVMR\text{\_}mean \end{array}$$

(2) $$\begin{array}{r} TRVI=IRT\text{\_}mean-AST\text{\_}mean \end{array}$$

(3) $$\begin{array}{r} AC=\frac{WDR\text{\_}MMS}{WDL\text{\_}MMS} \end{array}$$

#### 3.2.2. Stroop Switching Card Test Dataset

We used the SSCT dataset available on demand (see Data Availability section). A sample of 103 participants aged 15–75 years volunteered for the experiment. The battery consisted of standardized neuropsychological tests evaluating cognitive flexibility (TMT), inhibition (Stroop color and word test [SCWT]; Golden and Freshwater, 1978), the SSCT (Belghali and Decker, 2019), updating (forward and backward digit span test; Wechsler, 1955), and information speed processing (digit symbol substitution test [DSST]; Wechsler et al., 1997). A single testing session lasted for approximately 1 h, and each participant was tested individually. The dataset and the methodology of the study is described in Belghali et al. (2020). Below is a brief description of the dataset.

##### 3.2.2.1. Cognitive Flexibility

Cognitive flexibility is the mental ability to switch between thinking about multiple concepts simultaneously. It is based on executive functions that involve conscious changes in attention (cognitive shifting) and unconscious shifts of attention between tasks (task switching).

The TMT was used to assess flexibility. It is a neuropsychological test of visual attention and task switching. The subject connects 25 consecutive targets in a sequential order. TMT consists of two parts (A and B). In the first part, the targets are presented as numbers and the participant is required to connect them. In the second part, the participant is required to alternate between numbers and letters (i.e., 1-A-2-B-3-C, etc.). The time of completion for each part is recorded. The SSCT dataset contains the final outcome of the TMT test, which is measured as the Switch Score (SS) or *TMT_BA_Time*, which is the time delay between switching attention between numbers and letters (see Formula 5). Other studies have also used the ratio of performance (see Formula 6) based on evidence that the ratio of performance provides an index of EF; although the parts of the TMT differ in motor control and perceptual complexity (Arbuthnott and Frank, 2000). The TMT reflects cognitive abilities (visual-conceptual, visuospatial, and visual-motor tracking) as well as sustained attention and task alternation. The results predict physical impairment and mortality in older adults because poor cognitive function is associated with shorter life expectancy (Vazzana et al., 2010).

##### 3.2.2.2. Inhibition

Inhibition was assessed using the classical Stroop test and its modified forms.

The first test was the classical SCWT starting with two basic tasks: color naming (part A) and word reading (part B). The third task (part C) contains an interference condition whereby individuals are asked to name the ink color, which does not correspond to the written word (e.g., “yellow” written in green ink). The incongruity between the ink color and the meaning of the word causes a time delay when performing part C compared with A and B. The examiner records the completion times of each task (i.e., STROOP A, STROOP B, and STROOP C) and the total number of errors in part C. The interference score (IS) is the dependent variable of interest (see Formula 4).

The SSCT developed by Belghali is a modified version of the SCWT. In addition to the classic interference condition, it includes a switching condition, where subjects are instructed to act in different ways depending on where the words are printed. The instructions are to either read the conflicting words (e.g., if “blue” is written in another color, the individual is instructed to read “blue”) or name the incongruently colored ink (e.g., if “yellow” is written in green ink, the participant is instructed to say “green”). The main reasoning behind this task is that inhibition and switching share brain networks, notably the prefrontal network. Moreover, inhibition and switching have been considered two sides of the same coin (Mostofsky and Simmonds, 2008). Age-related decreases in response inhibition accounts for rising up of interference on Stroop tasks (Troyer et al., 2006). Older adults whose executive performance reduces within 1 year have shown larger switch discrepancy scores (i.e., the difference in performance between the SSCT performance and the classical Stroop task) compared with those whose executive performance remains stable (Fine et al., 2008).

The following outcomes of the SSCT were used:

1. RT (SSCT_TIME): the global RT to complete the SSCT.
2. *The total number of response errors* (SSCT_ERROR): reflects accuracy.
3. *The inverse efficiency score* (SSCT_IES) by Bruyer and Brysbaert (Bruyer and Brysbaert, 2011): reflects the RT of the correct responses and combines the proportion of errors and RT into one variable (see Formula 7).
  Responses are faster and more accurate when incongruent trials occur immediately after incongruent trials (*conflict resolution*) than when they occur after the congruent ones (*conflict adaptation*). Some studies have measured conflict resolution by the difference in response errors (i.e., accuracy) between incongruent and congruent trials and gauge conflict adaptation based on the response error difference between congruent trials following incongruent trials and incongruent trials following incongruent trials (Puccioni and Vallesi, 2012a,b). However, we used the following approach:
4. *Conflict resolution* (SSCT_Conflict_Resolution) was measured as the total number of response errors in incongruent trials that followed incongruent trials, which is inhibition without a change in congruence and refers to the ability to select relevant information while suppressing distracting information that is irrelevant to the current goal of the task. The subsequent tasks were congruent regarding the required response.
5. *Conflict adaptation* (SSCT_Conflict_Adaptation) was measured as the total number of response errors in congruent trials that followed incongruent trials, which is inhibition with a change in congruence and refers to the ability to adjust responses in accordance with the congruence of both current and previous trials.
6. *Inhibition and switching* (SSCT_I_S) is another metric of the conflict resolution process. In the SSCT, conflict resolution is applied in two ways. The first involves a cognitive sequence that involves inhibition exclusively without a change in congruence (e.g., naming an incongruent ink color preceded by an incongruent ink color). The second involves both inhibition and switching without a change in congruence (i.e., switching between calling the incongruent ink of colors and reading the words).
7. *Working memory updating* (SSCT_Updating) was measured by the total number of errors while classifying cards after each trial. It assessed inhibition only and inhibition with switching.

##### 3.2.2.3. Updating

Updating was assessed using the DSFBT, which is a widely-used neuropsychological test for short-term verbal memory and is a component of the Wechsler memory scale (Woods et al., 2011). DSFBT includes two sequences: forward and backward. For the forward one, the participant repeats a series of numbers presented by the examiner in the same order. In the backward sequence, the participant recalls the numbers in the reverse order. The length of the sequence increases in subsequent trials. Two trials are presented for each list length. Each trial starts with two digits until the limit in list length is reached (nine forward and eight backward). The examiner stops when the subject fails both trials of the same list length successively or when the maximal list length is reached. The dependent variable of interest (DIGIT_SPAN_FWBW) is the total number of lists reported correctly for both sequences.

##### 3.2.2.4. Information Speed Processing

Information speed processing assessed with the DSST, which is sensitive to many domains of cognitive dysfunction. It is also sensitive to changes in cognitive functioning across a wide range of clinical populations. Symbol-coding paradigms that are similar to the DSST are included as subtests in the Brief Assessment of Cognition in Schizophrenia and Repeatable Battery for the Assessment of Neuropsychological Status. However, DSST has low specificity for determining which cognitive domain is affected (Jaeger, 2018). Performance on the DSST can be affected by associative learning, motor speed, attention, visuoperceptual functions (e.g., scanning and ability to write or draw), executive functions of planning and strategizing, and working memory. The DSST consists of nine digit symbol pairs (e.g., 1/-, 2/~ 7/{, 8/X, 9/=), followed by a list of digits. Under each digit, the subject is required to write down the corresponding symbol as fast as possible. The number of correctly processed symbols within the allocated time (Processing_speed) is measured.

(4) $$\begin{array}{r} IS=STROOP\;C-\frac{STROOP\;A+STROOP\;B}{2} \end{array}$$

(5) $$\begin{array}{r} TMT\text{\_}BA\text{\_}TIME=TMT\;B-TMT\;A \end{array}$$

(6) $$\begin{array}{r} TMT\text{\_}BA\text{\_}RATIO=\frac{TMT\;B}{TMT\;A} \end{array}$$

(7) $$\begin{array}{r} SSCT\text{\_}IES=\frac{SSCT\text{\_}TIME}{1-SSCT\text{\_}ERROR} \end{array}$$

### 3.3. Preprocessing of Data

The POBA dataset consists of a list of deidentified subject records, with one patient per row, which are stored in a comma-separated value format file. To convert data into a format suitable for ML applications, several preprocessing steps are performed. We cleaned the data by removing missing, unknown, or inappropriate values. In 26% of cases, values for wrist dynamometry attributes (WDL_MMS, WDR_MMS, and AC) were missing. We generated the values of missing attributes by using a linear regression model, which was trained on the available data as predictors and missing attributes as outcome variables. Then, the value of the AC feature was calculated using Formula 3. Then the numerical variables were normalized by subtracting the mean value and scaling to the attribute variance.

#### 3.3.1. To Form Clusters and Groups of Participants

**Figure 1:**
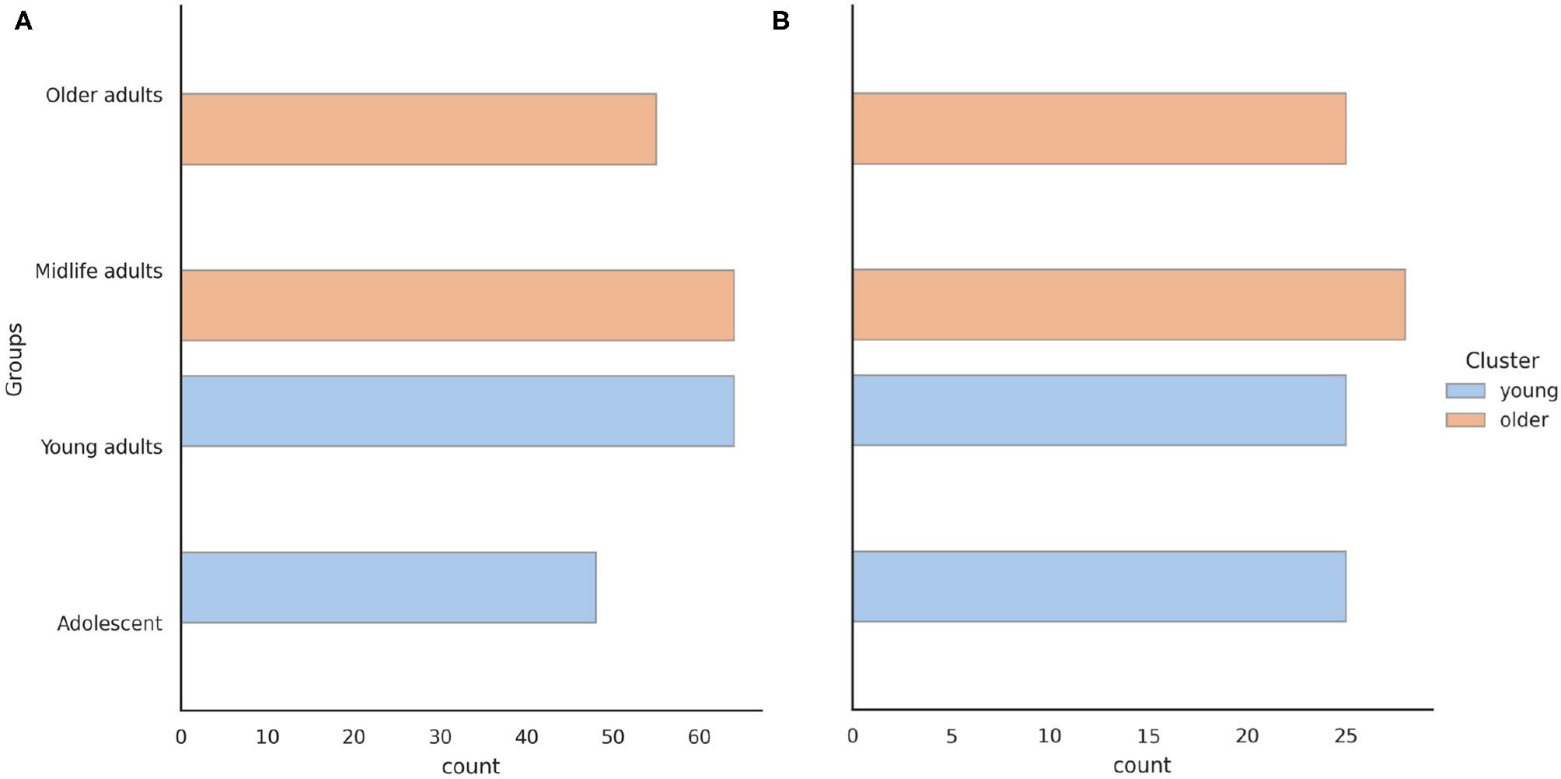
Age distribution of the **(A)** psychophysiological outcomes of brain atrophy and **(B)** stroop switching card test datasets.

To form clusters and groups of participants, we initially used four age groups. The range of years corresponding to each group was as follows: Adolescents were aged [0, 20) years, Young adults were aged [20, 40) years, Midlife adults were aged [40, 60) years, and Older adults were aged ≥ 60 years. As shown in Figure 1, the distribution of subjects by age group was similar. Subsequently we enlarged the groups into two major clusters. The clusters of the young (<40 years) and older (40 years and above) adults were almost balanced: 48.5/51.5%. While solving the last task, we excluded the demographic features from the dataset because they risked biasing the prediction.

### 3.4. Performance Evaluation Metrics

We used several objective measures to evaluate the performance of the clustering and classification methods. Confusion and error matrices were built for each predictive model to show how they distinguished between the younger and older classes. The ROC curve and AUC were used to evaluate the performance of the classifiers and summarize the trade-off between the true-positive (TPR) and false-positive rates (FPR), using different probability thresholds. The medical decision-making community has extensively published on the use of ROC graphs for the diagnostic testing (Fawcett, 2004) of balanced data (Saito and Rehmsmeier, 2015). Thus, we found that this metric was appropriate for our needs. We used: Here we use:

(8) $$\begin{array}{r} TPR\left(sensitivity\right)=\frac{TP}{TP+FN} \end{array}$$

(9) $$\begin{array}{r} TNR\left(specificity\right)=\frac{TN}{TN+FP} \end{array}$$

(10) $$\begin{array}{r} FPR=\frac{FP}{FP+TN}=1-specificity \end{array}$$

(11) $$\begin{array}{r} BAC\left(balanced\;accuracy\right)=\frac{Sensitivity+Specificity}{2} \end{array}$$

The overall accuracy of the model was defined as follows:

(12) $$\begin{array}{r} Accuracy=\frac{TP+TN}{TP+TN+FP+FN} \end{array}$$

where *TP,TN,FP*, and *FN* are true-positive, true-negative, false-positive, and false-negative values, respectively, representing the confusion matrix of the classification model.

All metrics were calculated for each fold separately, and averaged values were used as the final measure.

### 3.5. Hardware and Software

All experiments were conducted using a Linux Ubuntu 18.04 workstation with 24 CPU cores and two NVIDIA GeForce GTX 1080 Ti GPU with 11 GB GDDR5X memory each, using the Python programming language and its libraries for data processing, ML, and data visualization, such as scikit-learn, NumPy, Pandas, Matplotlib, Seaborn, and Plotly. For the POBA dataset collection, we used NS-Psychotest by Neurosoft.

## 4. Results

### 4.1. Association Between Test Performance and Age

**Figure 2:**
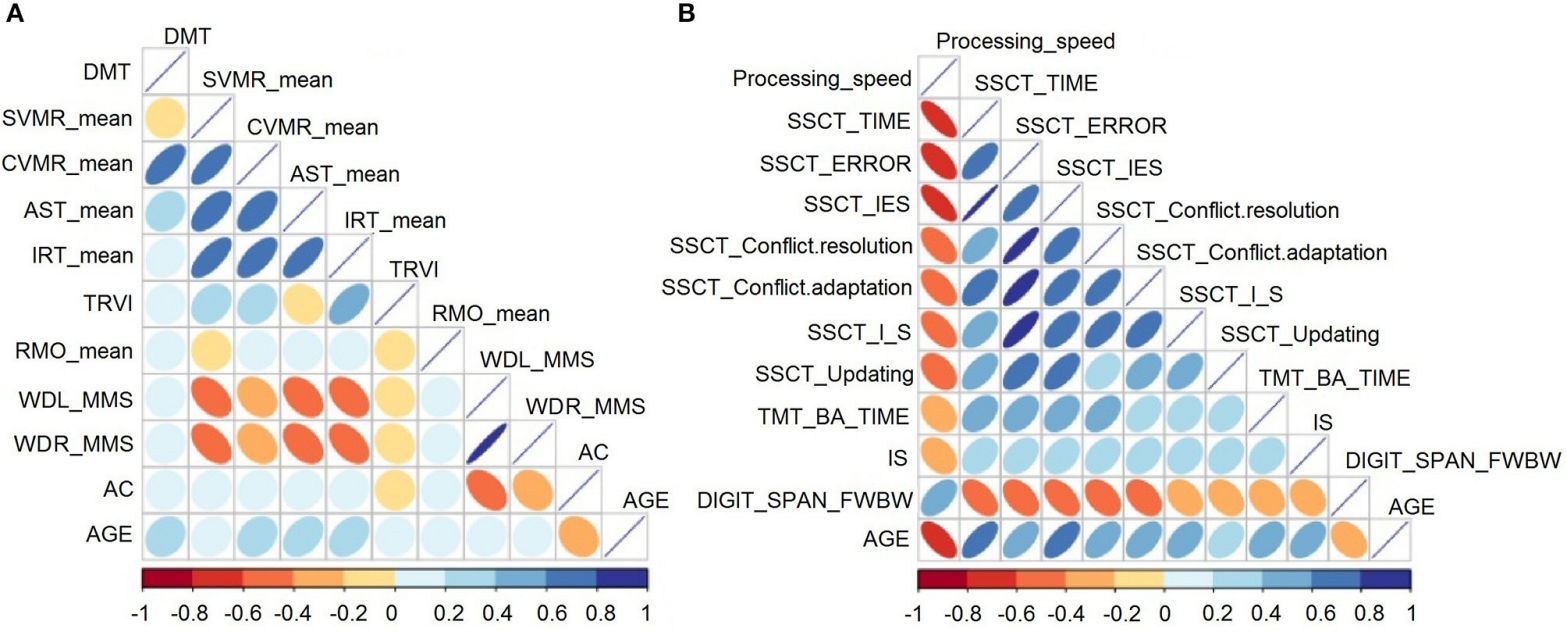
Correlation matrix heatmap for the **(A)** psychophysiological outcomes of brain atrophy and **(B)** stroop switching card test datasets.

Figure 2A describes the association between age and performance of participants for the PTs (i.e., the POBA dataset). Figure 2B shows the relationship between age and cognitive test performance (i.e., the SSCT dataset). The color intensity and size of the ellipses are proportional to the correlation coefficients.

*The analysis of the PTs* showed a positive correlation between age and all features except AC, which was negatively associated with age. Age was significantly associated with all psychophysiological parameters (*p* < 0.05) except for TRVI, RMO_mean, and wrist power.

*For the analysis of cognitive tests*, associations between test performance and age were significant and stronger compared with those between PT performance and age. Test output values increased with age because they reflected either the time taken to complete the task or the number of errors (inaccuracy). The exceptions were information speed processing from the DSST and accuracy in updating, reflected by the dependent variable of the DSFB test. Poorer test performance resulted in lower speed and accuracy estimates. Performance in the DSST and DSFB were negatively associated with age. All these changes demonstrate the inevitable decline in mental processes with age.

Apart from the correlations between age and basic neurophysiologic and cognitive functions, the diagrams showed strong associations of age with various attributes of behavioral test performance. Cognitive domains undergo age-related changes in parallel; therefore, such associations are not surprising. However, the onset and rate of change may differ between domains.

Tables 1, 2 show Pearson's correlation coefficients and p values for the association between age and psychophysiological test performance (the POBA dataset) and cognitive test performance (the SSCT dataset).

**Table 1:** Correlation matrix of psychophysiological tests performance and age.

**Feature**	**SVMR_mean**		**CVMR_mean**		**DMT**		**AST_mean**		**IRT_mean**		**TRVI**		**RMO_mean**		**AC**		**WDL_MMS**		**AGE**	
	***r***	***p*-value**	***r***	***p*-value**	**r**	***p*-value**	***r***	***p*-value**	***r***	***p*-value**	***r***	***p*-value**	***r***	***p*-value**	***r***	***p*-value**	***r***	***p*-value**	***r***	***p*-value**
Gender	0.095473	0.148039	0.122524	0.063012	0.077047	0.243452	0.158165	**0.016128**	0.180011	**0.006078**	0.068097	0.302743	-0.045266	0.493598	0.167460	**0.010791**	-0.482428	**<0.000001**	0.201525	**0.002084**
DMT	-0.000722	0.991298	0.671566	**<0.000001**	1.000000	**<0.000001**	0.205353	**0.001703**	0.198933	**0.002385**	0.039182	0.553503	0.089593	0.174768	0.053556	0.417856	0.045665	0.489790	0.203648	**0.001864**
SVMR_mean	1.000000	**<0.000001**	0.740460	**<0.000001**	-0.000722	0.991298	0.626474	**<0.000001**	0.716737	**<0.000001**	0.275004	**0.000022**	-0.009266	0.888610	0.110957	0.092481	-0.441085	**<0.000001**	0.137067	**0.037363**
CVMR_mean	0.740460	**<0.000001**	1.000000	**<0.000001**	0.671566	**<0.000001**	0.602201	**<0.000001**	0.664766	**<0.000001**	0.230098	**0.000422**	0.053350	0.419648	0.118208	0.072948	-0.296128	**0.000005**	0.238431	**0.000255**
AST_mean	0.626474	**<0.000001**	0.602201	**<0.000001**	0.205353	**0.001703**	1.000000	**<0.000001**	0.717224	**<0.000001**	-0.165172	**0.011934**	0.025443	0.700491	0.137768	**0.036392**	-0.425372	**<0.000001**	0.367574	**<0.000001**
IRT_mean	0.716737	**<0.000001**	0.664766	**<0.000001**	0.198933	**0.002385**	0.717224	**<0.000001**	1.000000	**<0.000001**	0.568806	**<0.000001**	0.009808	0.882141	0.105885	0.108472	-0.444672	**<0.000001**	0.361181	**<0.000001**
TRVI	0.275004	**0.000022**	0.230098	**0.000001**	0.039182	0.553503	-0.165172	**0.011934**	0.568806	**<0.000001**	1.000000	**<0.000001**	-0.016148	0.807138	-0.012742	0.847255	-0.127299	0.053341	0.077349	0.241606
RMO_mean	-0.009266	0.888610	0.053350	0.419648	0.089593	0.174768	0.025443	0.700491	0.009808	0.882141	-0.016148	0.807138	1.000000	**<0.000001**	0.052588	0.426334	0.075441	0.253450	0.040866	0.536581
WDL_MMS	-0.441085	**<0.000001**	-0.296128	**0.000005**	0.045665	0.489790	-0.425372	**<0.000001**	-0.444672	**<0.000001**	-0.127299	0.053341	0.075441	0.253450	-0.458617	**<0.000001**	1.000000	**<0.000001**	0.106160	0.107552
AC	0.110957	0.092481	0.118208	0.072948	0.053556	0.417856	0.137768	**<0.000001**	0.105885	0.108472	-0.012742	0.847255	0.052588	0.426334	1.000000	**<0.000001**	-0.458617	**<0.000001**	-0.218289	0.000838
AGE	0.137067	**0.037363**	0.238431	**0.000255**	0.203648	**0.001864**	0.367574	**<0.000001**	0.361181	**<0.000001**	0.077349	0.241606	0.040866	0.536581	-0.218289	**0.000838**	0.106160	0.107552	1.000000	**<0.000001**

*The significant associations between features are marked in bold*.

**Table 2:** Correlation matrix of cognitive test performance and age.

**Feature**	**Processing_speed**		**SSCT_TIME**		**Conflict resolution**		**Conflict adaptation**		**SSCT_I_S**		**SCCT_Updating**		**TMT_BA_TIME**		**IS**		**DIGIT_SPAN_FWBW**		**AGE**	
	***r***	***p*-value**	***r***	***p*-value**	***r***	***p*-value**	***r***	***p*-value**	***r***	***p*-value**	***r***	***p*-value**	***r***	***p*-value**	***r***	***p*-value**	***r***	***p*-value**	***r***	***p*-value**
Processing_ speed	1.000000	**< 0.000001**	-0.760452	**< 0.000001**	-0.562762	**< 0.000001**	-0.569408	**< 0.000001**	-0.567443	**< 0.000001**	-0.416929	**0.000012**	-0.308709	**0.001510**	-0.347707	**0.000320**	0.541204	**< 0.000001**	-0.641117	**< 0.000001**
SSCT_TIME	-0.760452	**< 0.000001**	1.000000	**< 0.000001**	0.597524	**< 0.000001**	0.611631	**< 0.000001**	0.538556	**< 0.000001**	0.588301	**< 0.000001**	0.406676	**0.000020**	0.343364	**0.000384**	-0.515086	**< 0.000001**	0.682432	**< 0.000001**
SSCT_ERROR	-0.651959	**< 0.000001**	0.697348	**< 0.000001**	0.904227	**< 0.000001**	0.832309	**< 0.000001**	0.836126	**< 0.000001**	0.605417	**< 0.000001**	0.448494	**0.000002**	0.391254	**0.000044**	-0.457402	**0.000001**	0.591469	**< 0.000001**
SSCT_IES	-0.738842	**< 0.000001**	0.985101	**< 0.000001**	0.667223	**< 0.000001**	0.662397	**< 0.000001**	0.600535	**< 0.000001**	0.643902	**< 0.000001**	0.442140	**0.000003**	0.349678	**0.000294**	-0.507645	**< 0.000001**	0.660753	**< 0.000001**
SSCT_Conflict resolution	-0.562762	**< 0.000001**	0.597524	**< 0.000001**	1.000000	**< 0.000001**	0.704722	**< 0.000001**	0.644165	**< 0.000001**	0.371008	**0.000114**	0.456961	**0.000001**	0.378832	**0.000079**	-0.409567	**0.000017**	0.543748	**< 0.000001**
SSCT_Conflict adaptation	-0.569408	**< 0.000001**	0.611631	**< 0.000001**	0.704722	**< 0.000001**	1.000000	**< 0.000001**	0.608635	**< 0.000001**	0.454229	**0.000001**	0.387844	**0.000052**	0.355932	**0.000224**	-0.417635	**0.000011**	0.536724	**< 0.000001**
SSCT_I_S	-0.567443	**< 0.000001**	0.538556	**< 0.000001**	0.644165	**< 0.000001**	0.608635	**< 0.000001**	1.000000	**< 0.000001**	0.516613	**< 0.000001**	0.254225	**0.009561**	0.245022	**0.012613**	-0.337418	**0.000491**	0.471337	**0.000001**
SCCT_Updating	-0.416929	**0.000012**	0.588301	**< 0.000001**	0.371008	**0.000114**	0.454229	**0.000001**	0.516613	**< 0.000001**	1.000000	**< 0.000001**	0.318142	**0.001057**	0.304634	**0.001756**	-0.351070	**0.000277**	0.385386	**0.000058**
TMT_BA_TIME	-0.308709	**0.001510**	0.406676	**0.000020**	0.456961	**0.000001**	0.387844	**0.000052**	0.254225	**0.009561**	0.318142	**0.001057**	1.000000	**< 0.000001**	0.269438	**0.005919**	-0.253960	**0.009638**	0.442843	**0.000003**
IS	-0.347707	**0.000320**	0.343364	**0.000384**	0.378832	**0.000079**	0.355932	**0.000224**	0.245022	**0.012613**	0.304634	**0.001756**	0.269438	**0.005919**	1.000000	**< 0.000001**	-0.262351	**0.007425**	0.483265	**< 0.000001**
DIGIT_SPAN _FWBW	0.541204	**< 0.000001**	-0.515086	**< 0.000001**	-0.409567	**0.000017**	-0.417635	**0.000011**	-0.337418	**0.000491**	-0.351070	**0.000277**	-0.253960	**0.009638**	-0.262351	**0.007425**	1.000000	**< 0.000001**	-0.352941	**0.000256**
AGE	-0.641117	**< 0.000001**	0.682432	**< 0.000001**	0.543748	**< 0.000001**	0.536724	**< 0.000001**	0.471337	**0.000001**	0.385386	**0.000058**	0.442843	**0.000003**	0.483265	**< 0.000001**	-0.352941	**0.000256**	1.000000	**< 0.000001**

*The significant associations between features are marked in bold*.

### 4.2. Lookup for the Onset of Psychophysiological and Cognitive Decline

Most dependent variables of the test batteries are represented by low values for high performance and vice versa. However, several dependent variables have lower values for poor performance, which include the muscle strength parameters and outputs of the DSST and DFBW tests. To maintain consistency in the diagrams, we reversed the values for subsequent analyses (i.e., 1/WDL_MMS, 1/Processing_speed, and 1/DIGIT_SPAN_FWBW).

**Figure 3:**
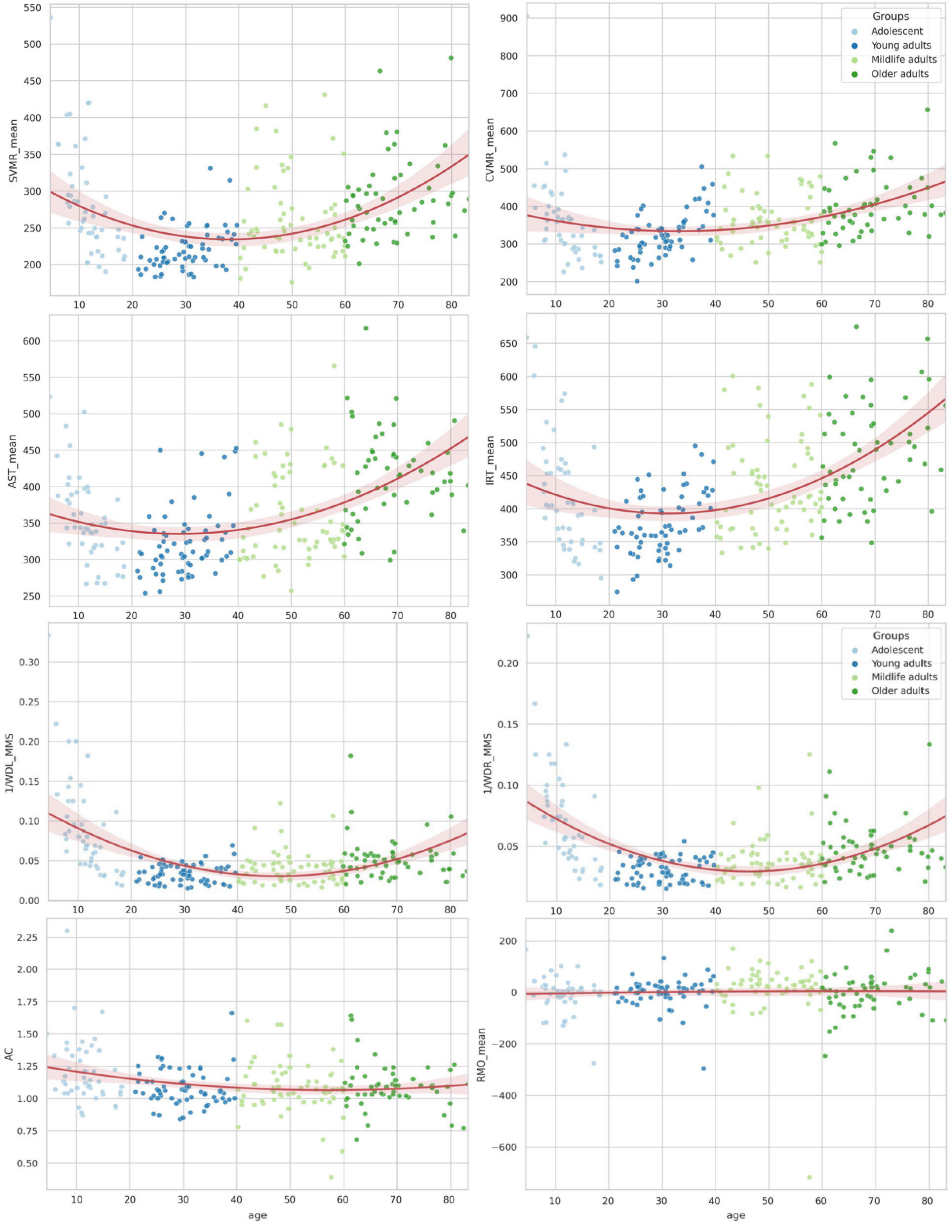
Distribution of reaction time attributes by age for psychophysiological tests and wrist dynamometry.

Table 3 shows the lifelong dynamics of PT performance. The minimal values of the variables in young adults indicated better performance than other groups across all PTs (Figure 3). The U-shaped curve of the minimal values in those aged 30–45 years was the common pattern for all age-related changes, except for those of AC and RMO_mean. AC values showed a slight descending trend toward 55 years and a similar ascending trend after 55 years. RMO_mean remained almost unchanged throughout life.

**Table 3:** Comparison of test performance by age group and sex for the psychophysiological outcomes of brain atrophy dataset.

**Test**	**Total**		**Female**	**Male**	**p_**1**__**−**__**2**_**
	**n**_****1****_ **= 231**		**n_**2**_ = 134 (58.01%)**	**n_**3**_ = 97 (41.99%)**	
*SVMR_mean*	260.51	[219.63–285.83]	265.33 ± 57.56	253.84 ± 61.31	**<0.0147**
Adolescent	282.03	[237.52–307.52]	290.9 ± 82.47	276.23 ± 61.5	0.433
Young adults	221.03	[201.72–235.03]	224.4 ± 25.96	216.69 ± 31.8	**<0.0454**
Midlife adult	259.76	[224.22–275.98]	269.65 ± 50.34	244.34 ± 59.48	**<0.0041**
Older adults	288.52	[254.25–304.47]	285.83 ± 50.36	295.71 ± 61.31	0.3423
*SVMR_variance*	69.88	[41.09–80.82]	70.82 ± 47.22	68.57 ± 48.48	0.3557
Adolescent	89.01	[48.0–90.89]	100.08 ± 82.66	81.75 ± 65.55	0.392
Young adults	49.41	[32.32–58.76]	49.26 ± 21.14	49.6 ± 23.9	0.3855
Midlife adult	67.69	[44.72–82.09]	71.5 ± 36.82	61.76 ± 35.31	0.1447
Older adults	79.54	[54.32–99.51]	75.68 ± 40.62	89.85 ± 47.01	0.0914
*CVMR_mean*	360.77	[307.45–395.57]	369.13 ± 81.17	349.22 ± 77.4	**<0.0259**
Adolescent	360.8	[291.42–392.68]	375.09 ± 142.16	351.43 ± 75.82	0.4497
Young adults	324.89	[289.33–346.97]	331.23 ± 47.76	316.73 ± 65.26	0.1236
Midlife adult	362.64	[316.64–393.8]	371.52 ± 60.98	348.79 ± 68.91	**<0.0466**
Older adults	400.32	[356.04–433.08]	398.07 ± 68.13	406.3 ± 80.78	0.4066
*CVMR_variance*	108.91	[70.7–118.64]	110.19 ± 79.9	107.14 ± 67.4	0.3801
Adolescent	121.55	[72.3–140.56]	102.73 ± 77.28	133.87 ± 102.52	*0.0523*
Young adults	91.82	[63.35–94.18]	96.51 ± 102.58	85.78 ± 34.53	0.2263
Midlife adult	92.65	[71.29–110.77]	98.4 ± 33.17	83.68 ± 22.95	0.0616
Older adults	136.69	[91.6–146.96]	137.54 ± 83.25	134.41 ± 45.39	0.1949
*AST_mean*	362.32	[311.05–412.35]	371.18 ± 68.9	350.09 ± 59.13	**<0.0114**
Adolescent	355.8	[319.45–389.67]	362.31 ± 69.28	351.54 ± 50.64	0.3839
Young adults	323.06	[293.45–339.52]	329.5 ± 52.82	314.77 ± 35.35	0.2735
Midlife adult	362.4	[317.05–418.52]	372.32 ± 63.1	346.92 ± 56.5	0.056
Older adults	413.62	[372.85–445.45]	411.79 ± 63.08	418.49 ± 54.49	0.2511
*AST_variance*	92.47	[53.55–115.6]	99.01 ± 60.08	83.42 ± 46.35	*0.0533*
Adolescent	89.45	[49.52–102.4]	90.95 ± 62.14	88.47 ± 54.17	0.3522
Young adults	67.67	[45.68–75.38]	74.02 ± 47.48	59.5 ± 18.24	0.4038
Midlife adult	89.68	[56.25–119.22]	95.88 ± 50.21	80.0 ± 36.0	0.117
Older adults	127.2	[74.45–168.0]	128.4 ± 65.63	124.0 ± 51.53	0.4962
*IRT_mean*	428.17	[368.15–471.85]	440.26 ± 77.99	411.48 ± 77.11	**<0.0021**
Adolescent	423.05	[351.78–462.75]	445.89 ± 102.78	408.09 ± 66.84	0.1365
Young adults	378.82	[344.05–416.9]	385.36 ± 43.16	370.42 ± 49.49	0.1365
Midlife adult	434.55	[377.52–477.58]	448.06 ± 65.5	413.47 ± 73.2	**<0.0125**
Older adults	482.65	[428.5–529.55]	479.39 ± 71.98	491.35 ± 82.16	0.374
*IRT_variance*	125.04	[76.75–156.85]	124.21 ± 64.11	126.19 ± 67.76	0.4734
Adolescent	147.01	[90.18–180.02]	150.61 ± 89.17	144.66 ± 70.13	0.4664
Young adults	91.84	[70.42–107.82]	88.71 ± 34.1	95.86 ± 46.43	0.4249
Midlife adult	112.04	[80.95–136.57]	114.84 ± 48.33	107.68 ± 50.54	0.1987
Older adults	159.64	[100.2–193.55]	152.78 ± 65.57	177.93 ± 79.24	0.1264
*RMO_mean*	0.32	[–18.5–31.35]	–2.72 ± 91.16	4.53 ± 58.05	0.4155
Adolescent	−8.99	[-22.48–16.67]	−11.31 ± 90.65	−7.48 ± 50.55	0.2882
Young adults	−2.14	[-12.38–20.95]	−1.96 ± 63.88	−2.37 ± 38.5	0.2624
Midlife adult	12.73	[-0.8–47.65]	−5.11 ± 124.22	40.56 ± 49.66	**<0.0341**
Older adults	−3.12	[-42.7–34.3]	2.99 ± 71.71	−19.44 ± 82.9	0.2886
*RMO_variance*	167.86	[84.7–224.35]	183.45 ± 107.07	146.33 ± 95.13	**<0.0014**
Adolescent	168.85	[80.0–216.25]	198.95 ± 123.68	149.12 ± 82.02	0.1147
Young adults	111.84	[62.53–137.75]	114.94 ± 70.91	107.85 ± 62.19	0.3251
Midlife adult	158.75	[88.48–214.9]	182.65 ± 96.42	121.47 ± 75.85	**<0.0006**
Older adults	242.81	[175.2–299.55]	238.53 ± 100.88	254.22 ± 115.11	0.3562
*1 / WDL_MMS*	0.05	[0.03–0.06]	0.06 ± 0.04	0.04 ± 0.04	**<0.001**
Adolescent	0.09	[0.05–0.11]	0.1 ± 0.07	0.08 ± 0.05	0.0714
Young adults	0.03	[0.02–0.04]	0.04 ± 0.01	0.03 ± 0.01	**<0.001**
Midlife adult	0.04	[0.03–0.05]	0.05 ± 0.02	0.02 ± 0.01	**<0.001**
Older adults	0.05	[0.04–0.06]	0.06 ± 0.03	0.03 ± 0.01	**<0.001**
*AC*	1.11	[1.01–1.19]	1.14 ± 0.19	1.07 ± 0.19	**<0.0001**
Adolescent	1.21	[1.05–1.34]	1.26 ± 0.2	1.18 ± 0.27	**<0.022**
Young adults	1.08	[1.0–1.15]	1.09 ± 0.15	1.07 ± 0.11	0.3598
Midlife adult	1.08	[0.99–1.18]	1.15 ± 0.2	0.98 ± 0.14	**<0.001**
Older adults	1.09	[1.02–1.14]	1.12 ± 0.18	1.02 ± 0.11	**<0.01**

*The significant differences between cohorts are marked in bold*.

The lifelong dynamics of cognitive test performance showed a different pattern. The performance metrics of the cognitive tests shared a similar overall trend, as seen in Table 4. Most values showed a rise from adolescence and an increase throughout life. However, several test estimates showed a small improvement in young adults, followed by steady worsening with age (e.g., SSCT_TIME, SSCT_IES, SSCT_Conflict adaptation, TMT_BA_TIME, and 1/DIGIT_SPAN_FWBW).

**Table 4:** Comparison of test performance in age groups with regard to gender in SSCT dataset.

**Test**	**Total**		**Female**	**Male**	**p_**1**__**−**__**2**_**
	**n**_****1****_ **= 103**		**n_**2**_ = 56(54.37%)**	**n_**3**_ = 47(45.63%)**	
*1 / Processing_speed*	0.03	[0.02–0.03]	0.03 ± 0.01	0.03 ± 0.01	0.1049
Adolescent	0.02	[0.02–0.02]	0.02 ± 0.0	0.02 ± 0.0	0.334
Young adults	0.02	[0.02–0.02]	0.02 ± 0.0	0.02 ± 0.01	0.079
Midlife adults	0.03	[0.02–0.05]	0.04 ± 0.02	0.03 ± 0.01	0.0513
Older adults	0.03	[0.02–0.04]	0.03 ± 0.01	0.03 ± 0.01	0.4537
*SSCT_TIME*	142.93	[81.5–178.0]	151.46 ± 92.75	132.77 ± 75.36	0.2301
Adolescent	84.24	[70.0–95.0]	84.08 ± 20.63	84.38 ± 23.92	*0.5*
Young adults	82.2	[69.0–95.0]	86.27 ± 21.76	76.1 ± 20.91	0.1728
Midlife adults	176.89	[134.75–201.5]	198.36 ± 75.35	155.43 ± 37.88	0.0805
Older adults	224.32	[150.0–269.0]	226.8 ± 101.94	220.6 ± 93.9	0.423
*SSCT_ERROR*	3.52	[0.0–6.0]	3.91 ± 3.97	3.06 ± 3.4	0.1537
Adolescent	1.32	[0.0–3.0]	1.17 ± 1.52	1.46 ± 1.45	0.3502
Young adults	1.88	[0.0–3.0]	2.27 ± 2.86	1.3 ± 1.9	0.2267
Midlife adults	3.79	[0.0–6.0]	4.79 ± 3.55	2.79 ± 2.62	**<0.0488**
Older adults	7.08	[4.0–11.0]	6.93 ± 4.31	7.3 ± 3.72	*0.5*
*SSCT_IES*	169.79	[84.35–213.03]	183.69 ± 141.44	153.23 ± 105.97	0.1983
Adolescent	87.74	[70.0–101.25]	87.39 ± 22.75	88.07 ± 25.07	0.4459
Young adults	87.36	[70.0–105.6]	92.41 ± 22.73	79.77 ± 24.2	0.106
Midlife adults	204.27	[144.0–232.29]	237.29 ± 108.98	171.24 ± 48.94	**<0.0468**
Older adults	295.66	[186.21–367.5]	301.99 ± 173.92	286.17 ± 137.78	0.4014
*SSCT_Conflict_* *resolution*	1.6	[0.0–3.0]	1.62 ± 1.82	1.57 ± 2.01	0.3547
Adolescent	0.52	[0.0–1.0]	0.42 ± 0.76	0.62 ± 0.74	0.2035
Young adults	0.96	[0.0–1.0]	1.13 ± 1.59	0.7 ± 1.19	0.3003
Midlife adults	1.68	[0.0–3.0]	2.0 ± 1.6	1.36 ± 1.63	0.1524
Older adults	3.24	[1.0–5.0]	2.73 ± 2.05	4.0 ± 2.28	0.098
*SSCT_Conflict_* *adaptation*	0.66	[0.0–1.0]	0.73 ± 0.81	0.57 ± 0.76	0.1575
Adolescent	0.36	[0.0–1.0]	0.33 ± 0.62	0.38 ± 0.49	0.3084
Young adults	0.2	[0.0–0.0]	0.27 ± 0.44	0.1 ± 0.3	0.1685
Midlife adults	0.64	[0.0–1.0]	0.86 ± 0.74	0.43 ± 0.73	*0.0548*
Older adults	1.44	[1.0–2.0]	1.4 ± 0.8	1.5 ± 0.67	0.4371
*SSCT_I_S*	0.91	[0.0–2.0]	1.05 ± 1.16	0.74 ± 0.91	0.1106
Adolescent	0.4	[0.0–1.0]	0.42 ± 0.64	0.38 ± 0.62	0.4604
Young adults	0.6	[0.0–1.0]	0.73 ± 1.0	0.4 ± 0.66	0.2136
Midlife adults	0.93	[0.0–2.0]	1.07 ± 0.96	0.79 ± 0.94	0.2107
Older adults	1.72	[1.0–3.0]	1.87 ± 1.31	1.5 ± 0.92	0.1632
*SSCT_Updating*	0.31	[0.0–0.0]	0.41 ± 0.77	0.19 ± 0.49	*0.0644*
Adolescent	0.04	[0.0–0.0]	0.0 ± 0.0	0.08 ± 0.27	0.1892
Young adults	0.12	[0.0–0.0]	0.13 ± 0.34	0.1 ± 0.3	0.4219
Midlife adults	0.39	[0.0–0.25]	0.5 ± 0.73	0.29 ± 0.7	0.1444
Older adults	0.68	[0.0–1.0]	0.93 ± 1.06	0.3 ± 0.46	0.0772
*TMT_BA_TIME*	56.46	[33.5–75.5]	56.04 ± 29.01	56.96 ± 35.51	0.4148
Adolescent	45.24	[33.0–58.0]	47.17 ± 13.89	43.46 ± 18.16	0.3315
Young adults	40.76	[24.0–52.0]	40.2 ± 18.9	41.6 ± 11.28	0.3488
Midlife adults	60.79	[43.75–86.5]	63.79 ± 32.27	57.79 ± 39.11	0.2675
Older adults	78.52	[58.0–107.0]	71.73 ± 32.3	88.7 ± 41.81	0.0785
*IS*	46.51	[29.5–58.75]	49.38 ± 23.23	43.11 ± 24.19	*0.0717*
Adolescent	29.3	[17.5–44.0]	29.96 ± 12.18	28.69 ± 18.97	0.4352
Young adults	40.42	[24.5–51.0]	46.73 ± 19.2	30.95 ± 13.91	**<0.0132**
Midlife adults	53.57	[42.75–63.88]	54.07 ± 18.95	53.07 ± 23.11	0.3312
Older adults	61.92	[45.0–76.5]	63.17 ± 25.94	60.05 ± 22.31	0.4889
*1/DIGIT_SPAN_FWBW*	0.06	[0.05–0.07]	0.06 ± 0.01	0.06 ± 0.01	0.1264
Adolescent	0.06	[0.05–0.06]	0.06 ± 0.01	0.06 ± 0.01	0.4649
Young adults	0.05	[0.04–0.06]	0.05 ± 0.01	0.05 ± 0.01	*0.0616*
Midlife adults	0.06	[0.06–0.07]	0.07 ± 0.01	0.06 ± 0.01	0.1368
Older adults	0.06	[0.05–0.07]	0.06 ± 0.01	0.06 ± 0.01	0.4096

*The significant differences between cohorts are marked in bold*.

**Figure 4:**
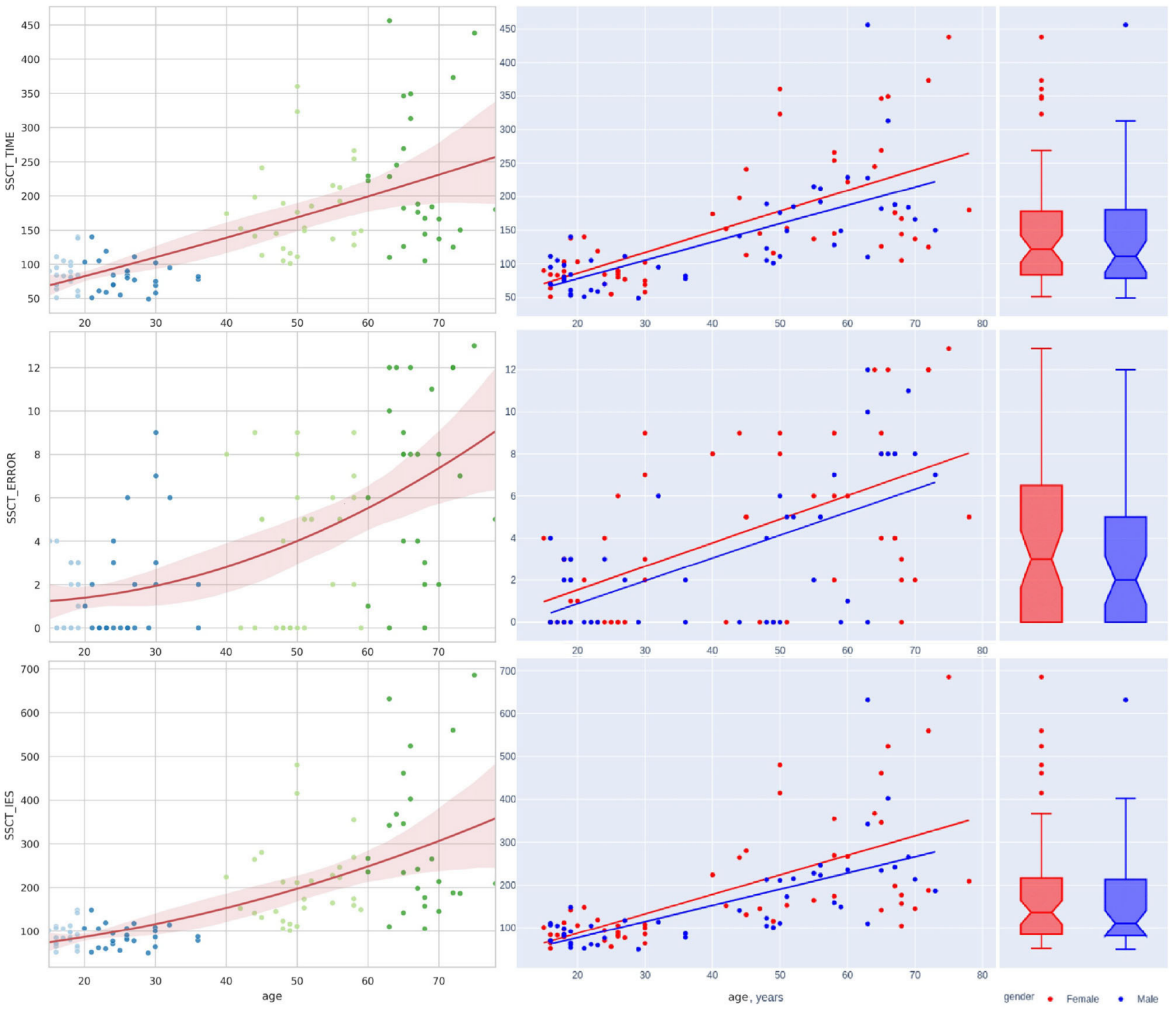
Distribution of reaction time and accuracy attributes in Stroop switching card test by age.

**Figure 5:**
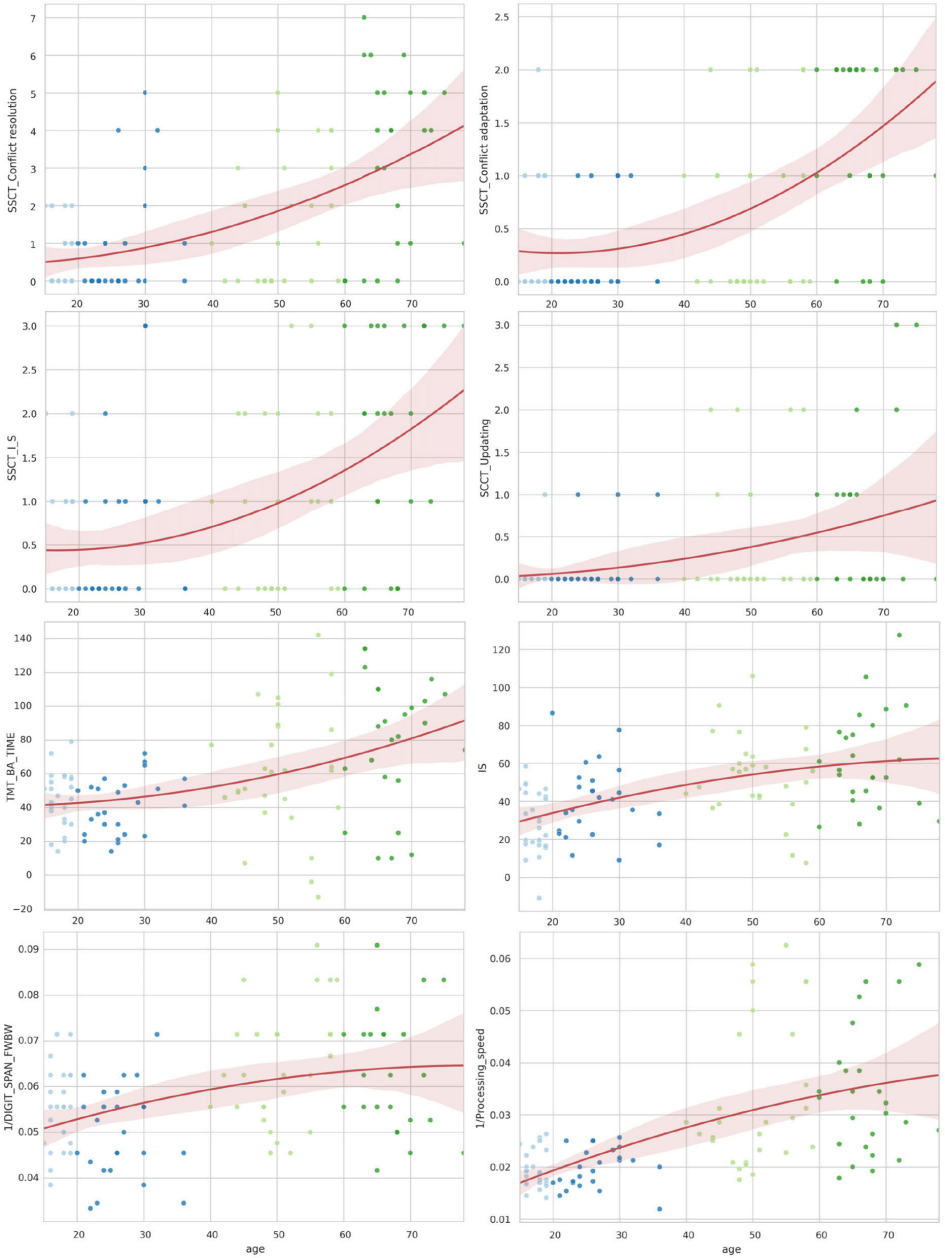
The distribution of the Stroop switching card test, trial making test, Stroop color and word test, digit span forward and backward test, and digit symbol substitution test by age.

Figures 4, 5 illustrate the data in table. Only the SSCT_Conflict adaptation and SSCT_I_S curves presented an optimal value in those aged over 25 years with the following worsening of the parameters. All other dependent variables of the cognitive tasks progressed steadily throughout life.

### 4.3. Sex Differences in Lifelong Test Performance

**Figure 6:**
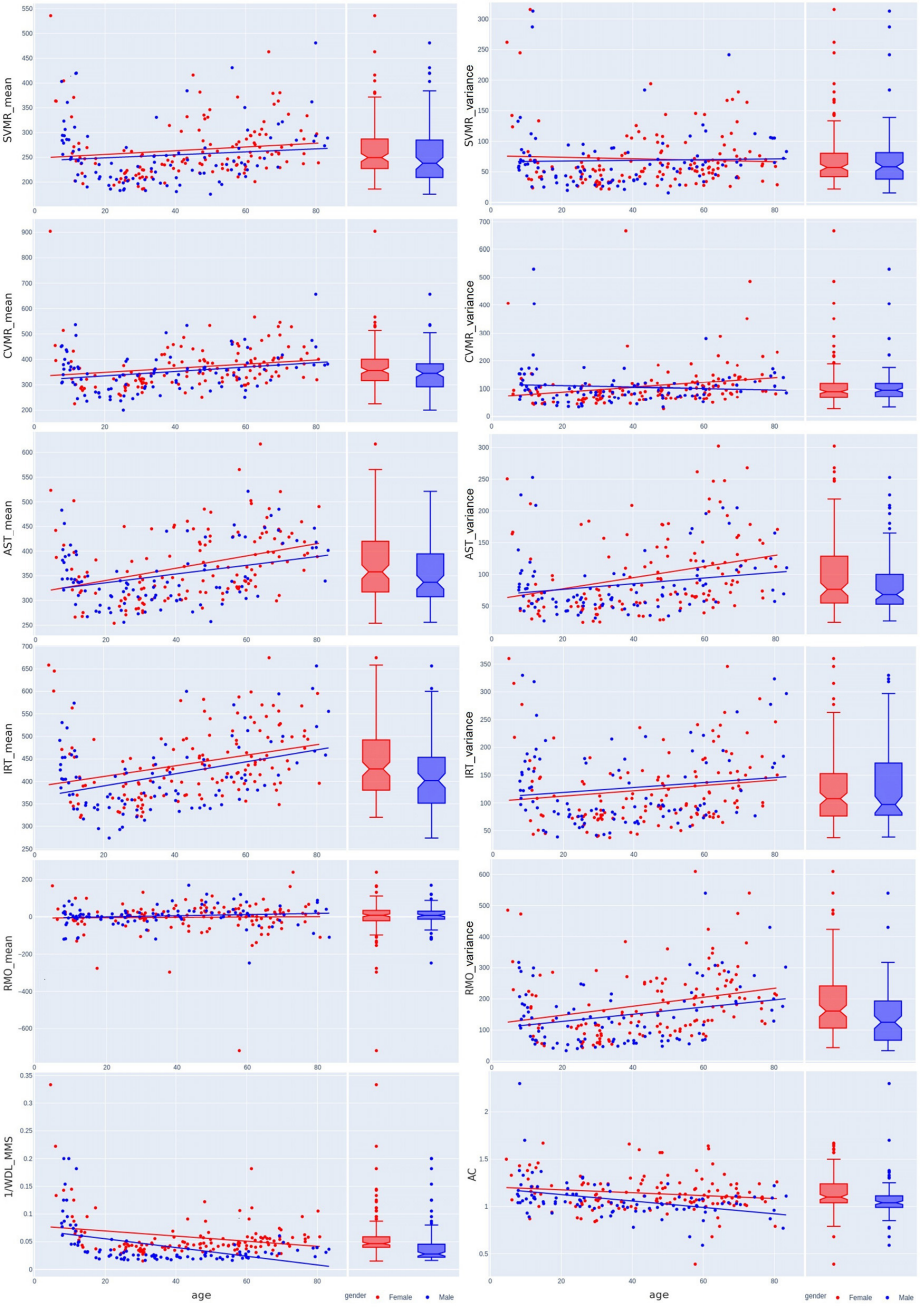
Sex-related differences in reaction time attributes and results of wrist dynamometry.

**Figure 7:**
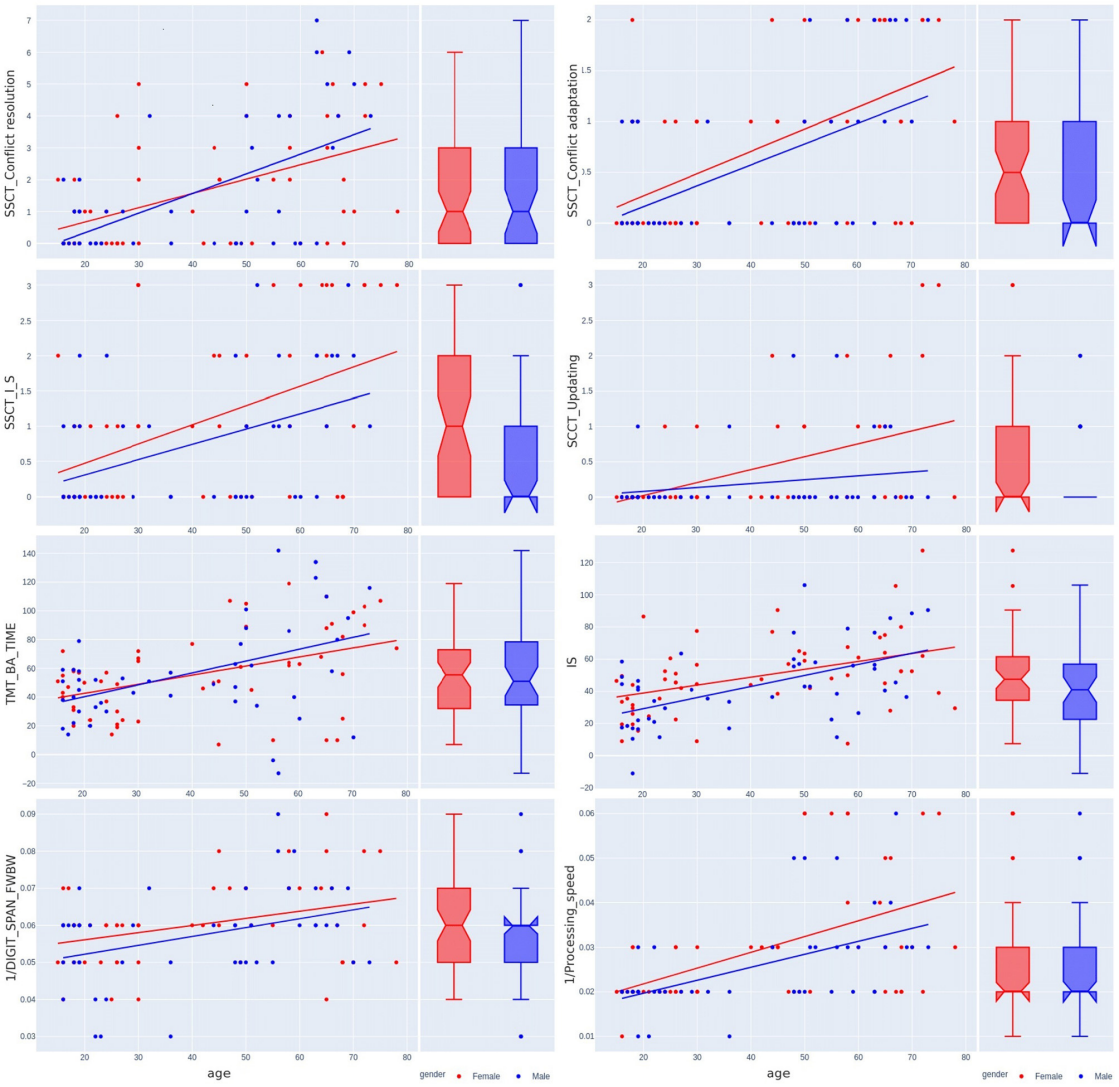
Sex-related differences in the Stroop switching card test, trial making test, Stroop color and word test, digit span forward and backward test, and digit symbol substitution test across the lifespan.

Table 3 shows the variance of PT performance by age and sex. From the averaged group data, men outperformed women in all PTs except for IRT_variance, which was similar across both sexes, with a slightly lower value in women (Figure 6). Table 4 and Figures 4, 7 show data of the cognitive test performance. No significant variance was related to sex.

Table 5 summarizes sex-specific lifelong changes of the variables. No significant differences were found among slopes or intercepts except for choice RT (CVMR_variance). Figure 6 shows that during adolescence, CVMR_variance remains unchanged throughout life in men. In contrast, in women, CVMR_variance increases with age. Figure 7 illustrates the different trends of changes with age for SSCT_Updating. In men, it remains relatively stable, whereas in women it increases. The significant difference in slope indicates different rates of deterioration between the sexes for this cognitive feature (see Table 5). There were no sex-related differences in the dynamics of age-related changes of psychophysiological or cognitive tests.

**Table 5:** Interaction coefficients for the comparison of intercepts and slopes for the psychophysiological outcomes of brain atrophy and Stroop switching card test datasets.

		**A comparison of the intercepts**		**A comparison of the slopes**	
**A cross-gender comparison**		***Estimate ±Std.Error***	***p-value***	***Estimate ±Std.Error***	***p-value***
SVMR	Mean	-5.5398 ± 17.0673	0.746	-0.0740 ± 0.3739	0.843
*in females vs. males*	Variance	-9.8509 ± 13.8649	0.478	0.1805 ± 0.3038	0.553
CVMR	Mean	-14.48187 ± 22.57078	0.5218	0.04636 ± 0.49450	0.9254
*in females vs. males*	Variance	42.6654 ± 21.431	**0.0477**	-1.0625 ± 0.4695	**0.02459**
AST	Mean	2.8253 ± 17.6758	0.873	-0.3607 ± 0.3873	0.353
*in females vs. males*	Variance	7.6835 ± 15.3078	0.616202	-0.4315 ± 0.3354	0.199539
IRT	Mean	-24.2166 ± 21.2315	0.25524	0.1578 ± 0.4652	0.73476
*in females vs. males*	Variance	7.51771 ± 18.87285	0.6908	-0.03684 ± 0.41348	0.9291
RMO	Mean	-0.04731 ± 22.90908	0.998	0.22319 ± 0.50191	0.657
*in females vs. males*	Variance	-14.3708 ± 28.6184	0.616047	-0.2828 ± 0.6270	0.652444
MMS	1/WDL	-0.0074758 ± 0.0103231	0.46970	0.0003275 ± 0.0002262	0.14892
*in females vs. males*	1/WDR	-0.0044287 ± 0.0074259	0.5515	-0.0002823 ± 0.0001627	0.0840
	AC	-0.012251 ± -0.012251	0.8198	-0.001863 ±0.001177	0.1148
SSCT	TIME	-0.9656 ± 28.6265	0.973	-0.3570 ± 0.6342	0.575
*in females vs. males*	ERROR	-0.591655 ± 1.382154	0.67	-0.003264 ± 0.030619	0.915
	IES	5.3695 ± 43.5334	0.902	-0.7681 ± 0.9644	0.428
	Conflict resolution	-0.67041 ± 0.73378	0.363	0.01670 ± 0.01626	0.307
	Conflict adaptation	-0.079453 ± 0.307563	0.797	-0.001345 ± 0.006813	0.844
	Updating	0.313096 ± 0.2566	0.2566	-0.012768 ± 0.006079	**0.0382**
	I_S	-0.056327 ± 0.426148	0.895	-0.005542 ± 0.009440	0.558
TMT_BA_TIME		-6.1996 ± 13.2474	0.640824	0.1950 ± 0.2935	0.507837
IS		-13.5758 ± 9.5060	0.15640	0.1958 ± 0.2106	0.35477
1/DIGIT_SPAN_FWBW		-4.820e-03 ± 5.060e-03	0.3432	4.676e-05 ± 1.121e-04	0.6775
1/Processing_speed		-8.548e-04 ± 4.835e-03	0.86	-6.251e-05 ± 1.071e-04	0.561

*The significant differences between intercepts and between slopes are marked in bold*.

### 4.4. Prediction of the Age Group Using Machine Learning

To estimate the onset of cognitive decline, we used cluster analysis. After assessing the outcome metrics of clustering into several groups, we obtained the best performance using two clusters when the cutoff value was set to 40 years of age (see Table 6). We achieved the best performance using the GenClus++ method (a combination of K-Means and the genetic algorithm). The misclassification of young participants was less frequent than that of older adults. This may account for the cumulative effect of individual lifestyle on cognitive status. Neurodevelopment in youth appears to be a more standardized process than brain aging of diverse origin, pace, and extent.

**Table 6:** Performance of clustering machine learning methods for the psychophysiological outcomes of brain atrophy and Stroop switching card test datasets assessed using the confusion matrix and prediction accuracy.

		**All features**						**Selected features**					
**Method**	**Class**	**POBA dataset**			**SSCT dataset**			**POBA dataset**			**SSCT dataset**		
		**ỹ**	**õ**	**A, %**	**ỹ**	**õ**	**A, %**	**ỹ**	**õ**	**A, %**	**ỹ**	**õ**	**A, %**
Simple K-Means Arthur and Vassilvitskii, 2006	Young	90	22	62.77	45	5	76.70	88	24	64.94	49	1	78.64
	Older	64	55		19	34		57	62		21	32	
Canopy McCallum et al., 2000	Young	87	25	64.50	36	14	71.84	89	23	63.2	50	0	84.41
	Older	57	62		15	38		62	57		14	39	
Expectation maximization Dempster et al., 1977	Young	82	30	65.37	40	10	76.70	83	29	66.67	49	1	**84.47**
	Older	50	69		21	32		48	71		17	36	
GenClus++ Islam et al., 2018	Young	87	25	67.53	48	2	77.67	87	25	**68.40**	49	1	82.52
	Older	50	69		21	32		48	71		17	36	

* Rows correspond to clusters; whereas columns correspond to values predicted by clustering method.

*ỹ,õ - number of predicted subjects belonging to the group young and old groups respectively*.

*A- the accuracy of correctly-predicted instances using the confusion matrix (see section 3.4)*.

*The largest accuracy value for each datasets is marked in bold*.

Initially, the clustering generated low prediction accuracy (68.4%). To improve the performance of clustering of the POBA dataset, we resorted to using the feature-selection method. The genetic algorithm returned the following list of features that maximized prediction accuracy: AST_mean, IRT_mean, SVMR_mean, and CVMR_mean (see Table 7). When we ran the information gain-based ranker for the SSCT dataset, we retrieved the following informative features: SSCT_TIME, SSCT_IES, Processing_speed, TMT_BA_TIME, and SSCT_ERROR. When we fed the unsupervised ML clustering models with the aforementioned features, the separability of the subjects by age group improved considerably.

**Table 7:** Features retrieved using the information gain-based ranker method.

**POBA dataset**		**SSCT dataset**	
**Attribute**	**Cognitive and neuropsychological features**	**Attribute**	**Cognitive and neuropsychological features**
AST_mean	Attention, information processing	SSCT_TIME	Information processing
IRT_mean	Attention, information processing	SSCT_IES	Updating, information processing
SVMR_mean	Information processing	Processing_speed	Information processing
CVMR_mean	Cognitive flexibility (switching), Information processing	TMT_BA_TIME	Cognitive flexibility, Information processing
RMO_mean	Neuropsychological stability	SSCT_ERROR	Accuracy
TRVI	Attention	IS	Information processing
DMT	Information processing	SSCT_Conflict_adaptation	Switching, inhibition
WDL_MMS	Muscle strength	SSCT_Conflict_resolution	Switching, inhibition
WDR_MMS	Muscle strength	DIGIT_SPAN_FWBW	Working memory updating
AC	Functional asymmetry	SSCT_I_S	Switching, inhibition
		SSCT_Updating	Working memory updating

To estimate the utility of a novel battery of tests for diagnosing age-related cognitive changes, we built an ML classification model, which identified the age group of participants as either below or above 40 years of age. If the prediction is reliable, it may reflect a subtle biomarker for accelerated aging (neurodegeneration) in those misclassified by the algorithm. A cognitive disorder may be diagnosed by estimating the gap between the chronological and predicted (biological) age. To make such predictions, a larger dataset is required in future studies using ML.

**Figure 8:**
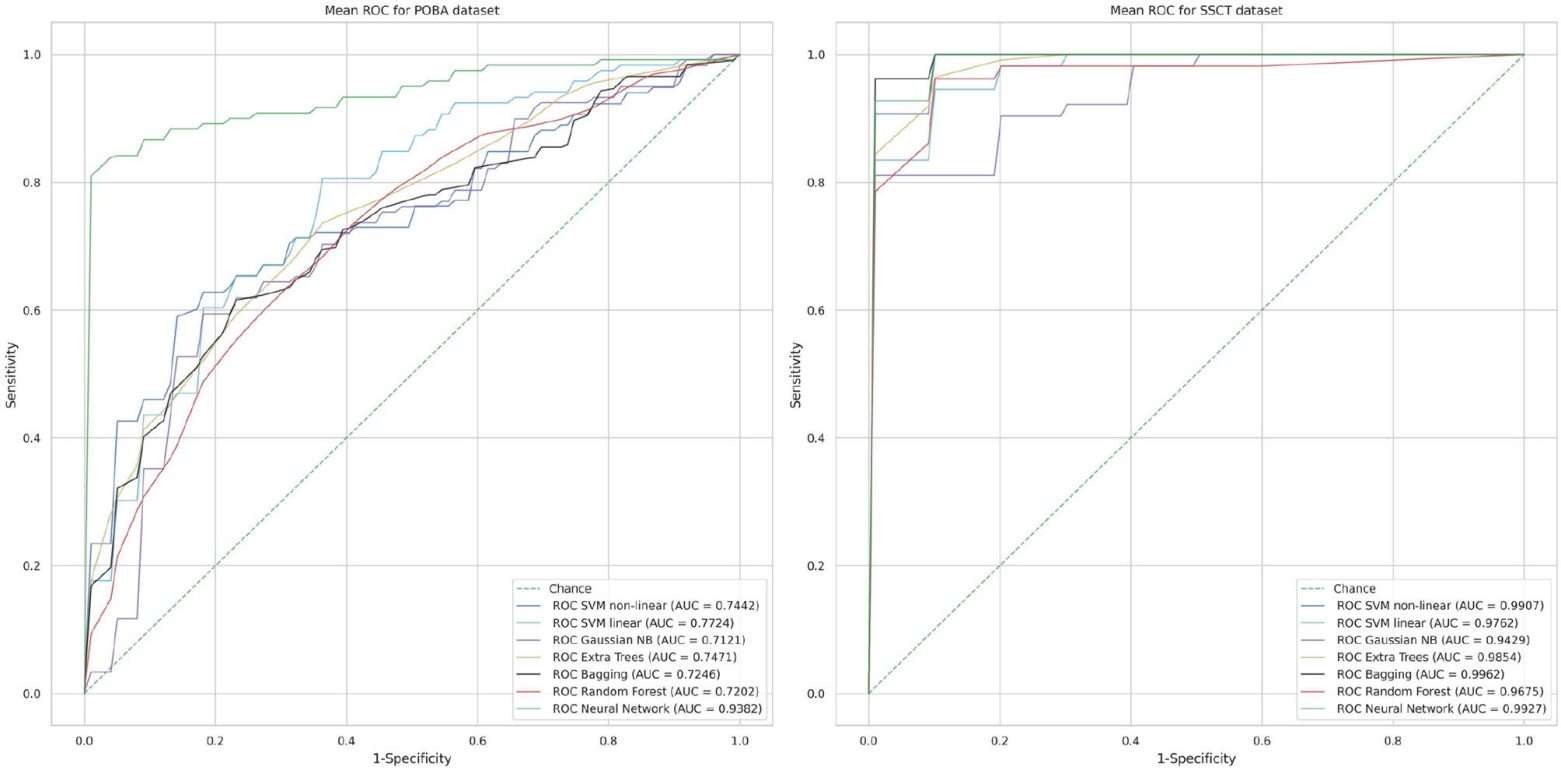
Classification performance of each method in terms of the mean receiver operating characteristic curve using a stratified five-fold cross-validation technique (area under the curve values close to 1 indicate a high level of diagnostic rate, whereas a value close to 0.5 shows poor performance).

In Table 8 methods known for their high performance in classifying numerical data are compared. Figure 8 shows the ROC curves and AUC values that represent the performance of classifiers in both datasets. The accuracy of age group prediction from cognitive test performance was higher than that of PT performance (maximal AUC for the SSCT dataset was 0.9962 vs. 0.9382 for the POBA dataset).

**Table 8:** Performance of the machine learning classification models for the psychophysiological outcomes of brain atrophy and Stroop switching card test datasets assessed by sensitivity, specificity, and area under the curve values.

	**Performance metrics**							
**Method**	**Class**	**POBA dataset**				**SSCT dataset**			
		**Sens**.	**Spec**.	**BAC**	**AUC**	**Sens**.	**Spec**.	**BAC**	**AUC**
SVM non-linear Platt, 1999	Young	0.66	0.7	0.68	0.7442	0.92	0.87	0.895	0.9907
	Older	0.7	0.66			0.87	0.92		
SVM linear Platt, 1999	Young	0.69	0.66	0.675	0.7724	0.86	0.91	0.885	0.9762
	Older	0.66	0.69			0.91	0.86		
Gaussian Naive Bayes John and Langley, 2013	Young	0.71	0.64	0.675	0.7121	0.88	0.83	0.855	0.9429
	Older	0.64	0.71			0.83	0.88		
Extra-trees classifier Geurts et al., 2006	Young	0.73	0.61	0.67	0.7471	0.93	0.9	0.915	0.9854
	Older	0.61	0.73			0.9	0.93		
Bagging meta-estimator Louppe and Geurts, 2012	Young	0.55	0.71	0.63	0.7246	0.94	0.89	0.915	**0.9962**
	Older	0.71	0.55			0.89	0.94		
Random Forest Breiman, 2001	Young	0.74	0.59	0.675	0.7202	0.9	0.87	0.885	0.9675
	Older	0.59	0.74			0.87	0.9		
Multi-layer Perceptron Glorot and Bengio, 2010	Young	0.97	0.86	0.915	**0.9382**	0.95	0.98	0.965	0.9927
	Older	0.86	0.97			0.98	0.95		

*The largest AUC value for each dataset is marked in bold*.

## 5. Discussion

### 5.1. Psychophysiological and Cognitive Performance With Age

#### 5.1.1. Dynamics of Psychophysiological Attributes Throughout Life

We used a battery of PTs comprising of cognitive domains and subdomains. SVMR reflected information processing, DMT represented task switching and inhibitory control, and IRT and AST measured attention. RT variability (RTV) across a set of trials reflected functional stability. To include the largest possible number of cognitive functions and components, we applied a comprehensive neurophysiological battery to study aging. However, we did not intend to explore specific cognitive subdomains, using specific tests. The idea was to study the basic neurophysiology that underlies complex behavior. The acquisition of visual-motor RT and its variance is straightforward, and the estimates provide an accurate physiologic assessment of individual neurodynamic properties.

##### 5.1.1.1. RT

In our study, RTs for visual-motor response, attention, switching, and inhibition were positively associated with age, which demonstrates age-related neurocognitive slowing. Measuring RT provides an insight into *information processing* that typically includes signal acquisition, decision-making, and response. There is a fundamental processing speed, i.e., the rate at which cognitive operations are executed. The definition suggests independence of higher-level cognitive operations from motor operations (Salthouse, 1996). Because the basic psychophysiological tasks that we used included motor responses, RTs may reflect EF rather than fundamental processing speed (Nilsson et al., 2014). The RTs that we measured were the aggregate output of a series of complex information-processing transactions that were initiated by the presentation of a stimulus and terminated by an overt response (Bashore et al., 1997).

##### 5.1.1.2. Choice RT

Choice RT (CRT) conveys information regarding concentration and processing speed. By subtracting simple RT from CRT, central processing time can be assessed, which accounts for 80% of age-related CRT slowing (Woods et al., 2015; Chintapalli and Romero-Ortuno, 2021). However, our data showed no significant difference between the rate of CVMR_mean and SVMR_mean slowing, which indicates impaired sensorimotor functions, rather than cognitive performance, and which at baseline explains the overall CRT slowing.

##### 5.1.1.3. RT Variability

Apart from RT, we also noticed age-related acceleration of its inconsistency (RTV) (Graveson et al., 2016). A psychophysiological explanation for this variability relates to fluctuations in executive control mechanisms. It may also reflect attentional sustainability (Bunce et al., 1993, 2004; West et al., 2002). Typical age-related neurocognitive slowing results in an increase in RTV across the lifespan (Hultsch et al., 2002), where RTV increases with response slowing (Haynes et al., 2017). This increase can be reduced by physical exercise (Bauermeister and Bunce, 2016; Haynes et al., 2017).

The effect of aging on RTV may increase with the complexity of a cognitive task (West et al., 2002; Bunce et al., 2004; Dixon et al., 2007), which we observed in the comparison between AST_variance and IRT_variance slopes and SVMR_variance and CVMR_variance slopes (see Figure 6 and Table 5).

There are varied opinions regarding the plausible metrics of RTV. In this study, we used standard deviation, which is in line with a systematic review that showed similar results independently, with different ways of assessing RTV (Haynes et al., 2017). However, another systematic review supported measures controlled for mean RT (e.g., coefficient of variation) (Graveson et al., 2016). The idea of adjusting RTV to RT is based on the high correlations between these variables. Therefore, age-related changes in RTV may reflect a general slowing of responses (Myerson et al., 2007). However, associations between RTV and clinical outcomes (e.g., dementia, falls, and death) suggest that neurocognitive variance is not simply related to general slowing (Graveson et al., 2016; Haynes et al., 2017).

##### 5.1.1.4. Attention

Rather than using choice and simple reactions exclusively, we employed attention study techniques (e.g., AST and IRT), which stemmed from the importance of evaluating attention. By driving goal-oriented behavior, attention determines the performance of any activity. Aging results in a reduced ability to concentrate on an object. Typical symptoms of advanced age neurodynamic disorders are talking around, inability to sustain attention, being easily distracted, and difficulty recalling information against a noisy background (Tanila et al., 1997). Age-related decline in attention may reduce the performance for simultaneously carrying out tasks. For example, RT increases in the elderly when they are asked to concurrently achieve postural stability. Furthermore, slower responses during a choice reaction test are a potential predictor of faster decline in mobility (Chintapalli and Romero-Ortuno, 2021). Although it is not well-understood, mobility and cognitive impairment accompany each other throughout life.

##### 5.1.1.5. Visual-Motor Task Performance

Although we used PTs with visual paradigms, it was still challenging to analyze performance of tasks that relied on visual sensory functioning because they comprise several components: sensory acquisition, cognitive appraisal, and processing. Each component may undergo age-related changes that result in the wrong behavior of the entire system. Despite being derived from sensory components, poor performance may be misinterpreted as a sign of cognitive decline. Increased age results in a decline in visual search performance; however, the reasons for this association remain unclear (Monge et al., 2017).

##### 5.1.1.6. Brain Functional Asymmetry

A possible reason for a negative association between AC and age is that the force of each wrist differs because of the asymmetrical atrophy of the brain during aging. This reduces the dominant position of the motor cortex of one side. Another reason is a reduction in white matter (WM) connectivity during life. Fiber loss in the corpus callosum disconnects the two hemispheres and reduces the suppression of the non-dominant hemisphere by the contralateral hemisphere (Teipel et al., 2009). If motor cortex activity becomes equal across hemispheres, AC will reduce.

#### 5.1.2. Lifelong Trend of Cognitive Performance

Tasks vary across POBA and SSCT studies depending on cognitive complexity. They range from those involving low cognitive demands (e.g., SVMR in the POBA dataset) to those requiring more complex cognition (e.g., cognitive tasks in the SSCT dataset). On average, the cognitive tasks comprised in the SSCT dataset are more demanding compared with the battery of PTs. This may also explain why decline starts early in life according to cognitive tests, whereas psychophysiological findings begin to worsen only from middle age.

##### 5.1.2.1. Task Switching and Conflict Resolution

Conflict resolution is an important cognitive ability that enables the suppression of automatic responses that may have been suitable previously but are inappropriate in a new context (Ho et al., 2019). Our findings on the lifelong dynamics of SSCT_Conflict_Resolution, SSCT_I_S, and 1/DIGIT_SPAN_FWBW are consistent with previous studies that showed poor dual-tasking abilities in older adults. The ability to switch between concurrent tasks is supported by executive control, which becomes weaker with age (Graveson et al., 2016). *Executive cognitive control* requires operations, such as conflict monitoring and response inhibition. Conflict monitoring is the evaluative component of cognitive control, as it detects the occurrence and level of conflict. The disproportionate deficits in inhibitory processing have shown to discriminate individuals with normal aging and MCI. *Conflict resolution* follows conflict monitoring and inhibits task-irrelevant responses and screens for task-relevant information (Cullen et al., 2007). The SSCT_I_S reliably reflects the ability to inhibit irrelevant responses and switch to a correct behavior (Belghali et al., 2020).

##### 5.1.2.2. Interference Score

Interference score is a dependent variable of SCWT, the diagnostic value of which is based on the *interference effect*. The effect leads to slower cognitive speed during incongruent trials compared with congruent trials. The effect is larger in cognitively-impaired people compared with cognitively-preserved individuals (Ho et al., 2019). In our study, IS increased steadily across the lifespan (see Figure 5).

##### 5.1.2.3. Processing Speed

In the battery of cognitive tests we used, DSST enabled us to assess information-processing speed, which showed significant age-related changes. Processing speed is particularly sensitive to age and mediates the decline in higher-order cognitive domains (Nilsson et al., 2014). An alternative point of view is that under appropriate control, processing speed accounts for most age-related differences in executive deficits (Verhaeghen and Cerella, 2002). For example, a study of three groups of participants, with mean ages of 22, 70, and 85 years, respectively, showed common perceptual and orienting attention patterns, and differences were observed for processing speed only (Muiños et al., 2016).

##### 5.1.2.4. Attention

Attention as a cognitive domain was also measured by the cognitive tests. This is because major complex activities require attentional resources. Multitasking suffers with advanced age because of the reduced ability to flexibly switch attention (known as *intellectual rigidity*). TMT reflects cognitive flexibility and involves attention. Thus, it is not surprising that its dependent variable, TMT_BA_TIME, decreased across the lifespan. Numerous studies have shown that aging results in disturbances of concentration and attention.

Similarly to attention, **working memory** is also involved in many cognitive tasks (e.g., the SSCT and DSST). Changes in the dependent variables of the tests (SSCT_Updating and Processing_speed) signify working memory decline with age.

### 5.2. Onset of Decline in Psychophysiological and Cognitive Performance

Cognitive decline may start at different ages. There is no common age of onset in any population. Previous studies have shown that the decline is already evident *at middle age*. The most affected functions are EF (Singh-Manoux et al., 2012) and processing speed (Salthouse, 2009; Zimprich and Mascherek, 2010). A recent study revealed that most age-related cognitive changes occur *at the age of 50–65 years*, with only a few age-related differences being evident *before the age of 50 years* (Ferreira et al., 2015). However, in healthy educated adults, some aspects of cognitive impairment have shown to start *during their 20s and 30s* (Salthouse, 2009). Some studies have suggested that crystallized intelligence continues to increase during adulthood, whereas decline in physiological cognitive functions (e.g., fluid intelligence, memory, and especially processing speed) starts earlier (Zimprich and Mascherek, 2010).

There is a considerable body of evidence that has implicated brain structural changes in age-related EF deficits. Below is the discussion of our findings of previous brain morphology studies, which give insight into the pathomorphological mechanisms of neurocognitive slowing.

#### 5.2.1. White Matter Changes and Psychophysiological Worsening

##### 5.2.1.1. Reaction Time and Processing Speed

RT and processing speed. RT estimates and RTV of the PT that we used followed a U-shaped function across the lifespan, which is consistent with the inverted U-shaped function of WM volume changes. WM volume increases until early middle age (35 years of age), which is followed by a period of stability, and finally an accelerated decline after late middle age (55–60 years of age). Furthermore, there is evidence that indices of WM integrity from diffusion tensor imaging (DTI) strongly correlate with processing speed. WM integrity changes start early in adulthood and show greater decline after the age of 60 (Ferreira et al., 2014; Nilsson et al., 2014).

A recent study found that age-related differences in two components of processing speed do not occur simultaneously. The cognitive component of processing speed integrates all the results. The slowing of the component occurs *before the age of 50 years*, whereas the motor component slows *during the age of 50–65 years* (Ferreira et al., 2015).

##### 5.2.1.2. RT Variance

The trial-to-trial volatility of RT across a task (RTV) is closely related to brain structural features and provides an insight into brain changes across the lifespan. RTV occurs because of the consequences of WM decline, such as less distinct cortical representations and increased neural noise. Independent of RT, RTV has been shown to be associated with the prevalence of WM lesions (hyperintensities on FLAIR). RTV is a measure of WM integrity alone (in DTI studies) and general neurological integrity at a biological level (Deary et al., 2006; Nilsson et al., 2014). Findings regarding the age at which RTV begins to increase are inconsistent. However, in line with our findings, age-related increases in variability are thought to begin *in middle age or earlier* (Haynes et al., 2017).

#### 5.2.2. Gray Matter Atrophy and Cognitive Decline

Age-related changes in gray matter (GM) also mediate cognitive performance across the lifespan (Ferreira et al., 2014). The shape of age-related variance of major cognitive test performance is close to a straight line, which is similar to the linear trend of decreases in GM volume across the lifespan. The neural centers that comprise GM are responsible for information synthesis (e.g., decision making) and establishing links (e.g., associative thinking and working memory).

##### 5.2.2.1. Inhibitory Process

A study using the Stroop test showed that age-related differences in cognitive inhibition occur *at age 50–65 years* alongside the onset of verbal fluency and premotor function decline (Ferreira et al., 2015). However, in our study, impairment started early in life (from adolescence), with a slow progression throughout life (see IS changes in Figure 5).

##### 5.2.2.2. Attention

Attention is an internal cognitive process for directing focus toward objects or locations while managing distractions. According to its sources, attention can be broken down into networks that carry out alerting and executive control functions. Several studies have suggested that age-related cognitive decline, especially EF, begins *early in life, childhood* (Finch, 2009; Salthouse, 2009), or *middle age* (Zhou et al., 2011). Age-related deterioration of the prefrontal lobe and dopaminergic system accounts for the impairment of executive attention after the age of 40 (Zhou et al., 2011). In a recent study, decline in the attentional domain was found to occur *during the transition from middle age (50 years) to old age (65 years)* (Ferreira et al., 2015).

Our data showed that performance in PTs that utilize attention begins to deteriorate at middle age. In contrast, performance in the cognitive tests used in the study shows an early onset of decline. Estimating the onset of decline in attention is difficult because of the lack of tasks that purely measure attention.

##### 5.2.2.3. Working Memory

EF decline in working memory occurs *before the age of 50*, which is reflected by changes in the manipulation of visual and verbal information. Changes in the latter are less prominent, as the visual modality is more demanding than the verbal modality, and more complex components are more vulnerable to change across the lifespan. Difficulties in verbal learning that start during middle age are likely to be more related to frontal lobe impairment than middle lobe impairment (Ferreira et al., 2015). The procedural memory component undergoes age-related changes that manifest as an increase in errors and time of execution, which are more related to inhibitory control (performed by the frontal lobe) and processing speed (Lezak et al., 2004; Ferreira et al., 2015).

It is difficult to estimate the onset of working memory decline using the tests that we included because the battery of tests used do not specifically measure working memory status.

### 5.3. Socio-Demographic Correlates in Age-Related Cognitive Impairment

Sociodemographic correlates, such as sex and education, can influence cognitive test performance. Literacy and higher educational level correlates with superior test performance on several cognitive domains (Ho et al., 2019). For our analysis of both datasets, we took into consideration the educational level of participants. For instance, we used literacy as an inclusion criterion. Moreover, we included adults who indicated that they completed a professional course after finishing general education. Another way to control for the level of intelligence is by considering the years of formal education. However, this has several limitations. Firstly, the intensity of training is not considered. Secondly, it does not reflect the level of intellectual activity after completion of formal education (e.g., postgraduate study). Furthermore, there is evidence that the effects of intelligence and formal education on the development of dementia differ. Schmand showed that a low reading test score predicted incident dementia better than a low level of education. Furthermore, the study found that a high occupational level had a protective effect (Schmand et al., 1997).

#### 5.3.1. Sex Differences in Age-Related Cognitive Impairment

Despite some variation in psychophysiological and cognitive findings across the lifespan, we found significant test performance differences related to sex. The linear trends of the age-related changes for choice RT and updating in the SSCT test (see Table 5) had significantly different slopes. However, we did not observe significant sex differences in the lifelong dynamics of major test estimates. There is currently limited agreement in the literature on sex differences. Longitudinal studies comparing changes in cognitive function and probability of Alzheimer's dementia in men and women have revealed confronting results (Barnes et al., 2003).

### 5.4. Identification of Accelerated Decline

The idea of studying EF in normal aging was motivated by the increasing evidence that deficits in certain EFs may arise at early stages of neurodegenerative disease (Ho et al., 2019). The most commonly-documented cognitive changes associated with old age are decline in memory, attention, and speed of processing of incoming information. All components of processing, from stimulus acquisition to response execution, decline with age. There is no strong consensus regarding whether the rate of decline is a process specific or common across all components (Bashore et al., 1997). Previous studies have shown that a set of influences on information-processing speed occur with advancing age. The best way to characterize these influences is by combining a variety of processing speed measures, which may be acquired from different tasks or acquisition methods (e.g., latency of evoked potentials and RT) (Bashore et al., 1997).

In our study, the linear trendlines for the variance of test estimates across the lifespan represent tendencies toward decline of functioning, specifically for information processing, attention, and switching. They all followed a common trend with a similar rate of progression. The scatterplots indicate that age-related neurocognitive slowing is an unavoidable process that occurs at a permanent rate.

The graphs show that age-related decline in functioning does not separate the population into obvious cohorts that can be easily observed in scatterplots. Visually, we could not estimate a threshold value that would indicate the onset of cognitive decline. Nevertheless, the results obtained are promising. For now, the classification algorithms can be used for screening purposes (see section 5.4.2). The short acquisition time allows testing of each patient for signs of enforced brain aging. Future studies of patients with dementia using the same PTs will provide further support for our findings. Using a larger sample size will improve the performance metrics so that the battery of PTs will be a reliable predictor of the age group. Although, cognitive monitoring is not a replacement for a thorough neuropsychological assessment, its use as a supplement may provide indices of key cognitive domains during a brief consultation (Ho et al., 2019).

#### 5.4.1. The Separability of Data and Onset of Decline

To improve prediction accuracy, we used feature selection. We expected that the highly-prevalent decline in some cognitive domains would be largely responsible for age-related functional fading. The time estimates for the attention study technique and motor-visual reaction tests were ranked the highest. All selected features reflected information-processing speed. Additionally, other cognitive domains and subdomains (e.g., attention and task switching) were involved. This is relevant to the recently-formulated assumption that slowing of functioning is a major outcome of aging. The dependent variables that were derived from studies of attention (TRVI), motor reaction (DMT), and RMO_mean had a value output of zero. From the perspective of the ranking method, these features can be considered as redundant because they do not provide additional information for the final model decision.

#### 5.4.2. Reflection of Age-Related Cognitive Changes With the Psychophysiological Tests

Multilayer perceptron or traditional fully-connected three-layer NN models show significantly higher AUC values (89.6%) compared with other methods using the POBA dataset. The NN is a model that mimics the behavior of data and finds hidden patterns within it. The sensitivity and specificity of the young class were retrieved at 89 and 81%, respectively. The model was more sensitive to the young class than to the older class, which is in line with the trend observed in our assessment of the separability of the data.

Our results demonstrate that cognitive tests and PTs may serve a diagnostic purpose as a screening tool. They are rapid, standardized, fully automated, easy to administer, highly reproducible, and have sufficient sensitivity and specificity (see section 5.4.2).

## 6. Strengths and Limitations of the Study

A limitation of the study is its cross-sectional design. This implies that participants of different ages were born and raised at a different time. Thus, the Flynn or antiFlynn effect may have an influence, which is the change in intelligence test scores across generations, and it may influence mean RTs (Woodley et al., 2013). Such effects have been reported during previous decades in various countries (Woodley, 2012). However, there is increasing evidence that the effect is due to changes in test-taking behavior over time rather than significant variability in intelligence (Must and Must, 2013).

Another limitation of the study is that the datasets we used were not completely comparable, as they were acquired within the last 5 years in different countries. However, the last decade of research has shown that cognitive differences between countries are becoming smaller (Meisenberg and Woodley, 2013), which reduces the potential differences between societies and partly overcomes the limitation of this study.

In contrast to studies that used no specific criterion for normative performance, we proposed an approach that may have clinical utility. The ML classification algorithm may serve as a reliable tool for detecting individuals with accelerated cognitive impairment. If the algorithm misclassifies a participant into an incorrect age group, the individual may be considered at risk of cognitive deterioration. This potential application of our approach for clinical purposes is a strength of the study. To implement the classifier in practice, the study will need to be extended in a larger sample of healthy participants. Further research is required to investigate the dynamics of the identified measures in normal and pathological-aging populations.

Comprehensive psychophysiological assessment and detailed analysis of cognitive functions using ML-based modeling allowed us to detect early cognitive decline. Our study is one of few that have explored a broad variety of cognitive measures in a cohort of young and middle-aged adults. Knowledge of the early stages of normal aging will facilitate early advanced diagnostics and prevention of pathological aging.

## 7. Conclusion

- The study introduced the concept of a predicted “cognitive” age that can be forecast from a set of tests and compared with the chronological age. In cases where there is a significant difference between the predicted and actual age, the participant may be considered susceptible to accelerated brain aging. This will allow the individual to undergo advanced diagnostic procedures and follow-up examinations.
- In our study all RT and variance estimates followed a U-shaped function across the lifespan, which reflected the known inverted U-shaped function of WM volume changes, with optimal values observed in early middle age (35 years), followed by a period of stability, and accelerated decline after late middle age (55–60 years). The shape of the age-related variance of the major cognitive test performance was close to a straight line, which was similar to the linear trend of the decrease in GM volume across the lifespan. The neural centers comprising GM are responsible for information synthesis (e.g., decision making) and establishing links (e.g., associative thinking and working memory).
- Overall, the battery of cognitive tasks we used was more demanding compared with the PTs, which may explain why the analysis of the cognitive tests showed a decline starting early in life. In contrast, the psychophysiological findings (simple and complex RT and its variance across trials) suggested that the onset of functional decline occurred at middle age.
- Our study suggested that cognitive aging results from the convergence of several processes described in recent findings. These processes were a decline in EF, overall cognitive slowing, and impairment in visual processing. The tests we used may serve a diagnostic purpose as a screening tool for early neurocognitive slowing. The batteries may be used as subtle biomarkers of neurodegeneration for individuals who are misclassified by the algorithm.
- The study did not show considerable sex differences in the lifelong dynamics of major test estimates, except for choice RT and updating in the SSCT test that showed a significantly-faster decline in women than in men.
- The performance of the classification model to identify the subjects' age group was promising. The sensitivity and specificity of the identification of the young class were 97 and 86%, using the PTs. The metrics of the cognitive tests were 95 and 98%, respectively. We observed better performance of the ML algorithms with the cognitive tests than the PTs as predictors (balanced accuracy was 96.5 vs. 94%, respectively), which is because of the linear change in cognitive estimates compared with the U-shaped change of the lifelong neurophysiological dynamics.
- ML models can be designed and utilized as a computer-aided detector of neurocognitive decline. Our study showed great promise for the use of classification models as predictors of age-related changes. Our results encourage us to explore a combination of tests from the battery to derive a more reliable set of tests based on performance metrics. Moreover, further investigations of other cognitive and PTs are warranted.
- Future research is required to improve the performance characteristics of the ML model by using a larger sample size and an enriched test dataset that includes patients with dementia.

## Data Availability Statement

The datasets presented in this study can be found in online repositories. The names of the repository/repositories and accession number(s) can be found below: The datasets generated for this study are available on request at the site of Big Data Analytics Center (BIDAC) at https://bi-dac.com.

## Ethics Statement

The studies involving human participants were reviewed and approved by United Arab Emirates University Human Research Ethics Committee (Notice Number: ERH_2019_4006 19_11) and CERSTAPS (Ethical Committee of Sport and Physical Activities Research (Notice Number: 2016-26-04-13). Written informed consent to participate in this study was provided by the participants' legal guardian/next of kin.

## Author Contributions

YS formulated the objectives, collected POBA dataset, and wrote the manuscript. TH did machine learning, formulated the methodology, and prepared graphs and tables. IC constructed the batteries of tests. KG, NZ, and TA contributed to literature review and data analysis. GB and ML supervised the research and formulated the conclusion. MB constructed the test battery, collected the SSCT dataset, and participated in manuscript writing.

## Conflict of Interest

The authors declare that the research was conducted in the absence of any commercial or financial relationships that could be construed as a potential conflict of interest.

## Abbreviations

AC: asymmetry coefficient
AUC: area under the curve
BAC: balanced accuracy
CVMR: complex visual-motor reaction
CRT: choice reaction time
DMT: decision-making time
DSFBT: digit span forward and backward test
DSST: digit symbol substitution test
DTI: diffusion tensor imaging
EF: executive functioning
EFT: executive functioning test
FN: false-negative values
FP: false-positive values
GM: gray matter
LOWESS: locally weighted scatterplot smoothing
ML: machine learning
NN: neural network
OLS: ordinary least squares
POBA: psychophysiological outcomes of brain atrophy
PS: psychophysiological status
PT: psychophysiological test
ROC: receiver operating characteristic
RT: reaction time
RTV: reaction time variability
SCWT: Stroop Color and Word Test
SSCT: Stroop Switching Card Test
SVMR: simple visual-motor reaction
TRVI: the time delay in responding to the targeted stimulus because of visual interfering objects
TN: true negative values
TP: true positive values
WM: white matter.
